# Preferred Endocytosis of Amyloid Precursor Protein from Cholesterol-Enriched Lipid Raft Microdomains

**DOI:** 10.3390/molecules25235490

**Published:** 2020-11-24

**Authors:** Yoon Young Cho, Oh-Hoon Kwon, Sungkwon Chung

**Affiliations:** Department of Physiology, Sungkyunkwan University School of Medicine, Suwon 16419, Korea; myjubilate@skku.edu (Y.Y.C.); jxstigma@skku.edu (O.-H.K.)

**Keywords:** Alzheimer’s disease, amyloid precursor protein (APP), cholesterol, lipid raft, endocytosis, raft-dependent APP endocytosis, drug target

## Abstract

Amyloid precursor protein (APP) at the plasma membrane is internalized via endocytosis and delivered to endo/lysosomes, where neurotoxic amyloid-β (Aβ) is produced via β-, γ-secretases. Hence, endocytosis plays a key role in the processing of APP and subsequent Aβ generation. β-, γ-secretases as well as APP are localized in cholesterol-enriched lipid raft microdomains. However, it is still unclear whether lipid rafts are the site where APP undergoes endocytosis and whether cholesterol levels affect this process. In this study, we found that localization of APP in lipid rafts was increased by elevated cholesterol level. We also showed that increasing or decreasing cholesterol levels increased or decreased APP endocytosis, respectively. When we labeled cell surface APP, APP localized in lipid rafts preferentially underwent endocytosis compared to nonraft-localized APP. In addition, APP endocytosis from lipid rafts was regulated by cholesterol levels. Our results demonstrate for the first time that cholesterol levels regulate the localization of APP in lipid rafts affecting raft-dependent APP endocytosis. Thus, regulating the microdomain localization of APP could offer a new therapeutic strategy for Alzheimer’s disease.

## 1. Introduction

Alzheimer’s disease (AD) is a progressive and irreversible neurodegenerative disease which is the most prevalent form of dementia [1]. The hallmarks of AD pathogenesis are the extracellular deposition of senile plaques and the presence of intracellular neurofibrillary tangles, which lead to severe neuronal atrophy and ultimately, death. Elevated levels and accumulation of cerebral β-amyloid (Aβ) peptides are the dominant pathological factor in the formation of senile plaques. Aβ is a byproduct of the sequential proteolytic cleavage of amyloid precursor protein (APP) by membrane bound β- and γ-secretases [2,3,4]. APP at the plasma membrane is internalized via endocytosis and delivered to early endosomes and lysosomes, where Aβ is produced. Therefore, APP is more likely to become accessible to β- and γ-secretases when the rate of APP endocytosis is increased, resulting in elevated production of Aβ [5,6]. Hence, the regulation of endocytic pathways plays a key role in the trafficking and processing of APP and subsequent Aβ generation.

Cholesterol is an essential component of the plasma membrane and has a number of physiological functions, among which is the regulation of endocytosis and exocytosis [7,8,9]. Therefore, cholesterol has been extensively implicated in the regulation of cellular APP processing, contributing to the development of AD [10,11,12,13,14,15,16]. Furthermore, lipid dyshomeostasis, including elevated cholesterol levels, is a key participant in the pathogenesis of AD [17,18,19,20,21,22,23]. Lipid raft microdomains, enriched with cholesterol and sphingolipids, are considered as cellular processing platforms for various cell signaling and protein–protein interactions [24,25,26,27]. Substantial evidence supports the importance of lipid raft microdomains in APP processing and Aβ production. It is widely believed that β- and γ-secretases as well as their substrate APP are localized in lipid raft microdomains [28,29,30,31]. In accordance with these reports, the increased membrane cholesterol induces APP endocytosis and Aβ generation [32,33,34,35,36,37,38]. Despite these efforts, the effect of increasing cholesterol level on APP endocytosis is not understood due to technical limitations. In the present study, we demonstrated that modulating cellular cholesterol levels redistributed surface APP between lipid rafts and nonrafts by using confocal microscopy and biotinylation method. By combining biotinylation and lipid raft fractionation methods, we also showed for the first time that APP localized in lipid rafts preferentially undergoes endocytosis compared to APP in nonrafts. Thus, our findings provide compelling evidence for the involvement of cholesterol-enriched lipid raft microdomains in APP endocytosis and Aβ production.

## 2. Results

### 2.1. Cholesterol Levels Regulate the Localization of Cell Surface APP in Lipid Raft Microdomains

**Figure 1:**
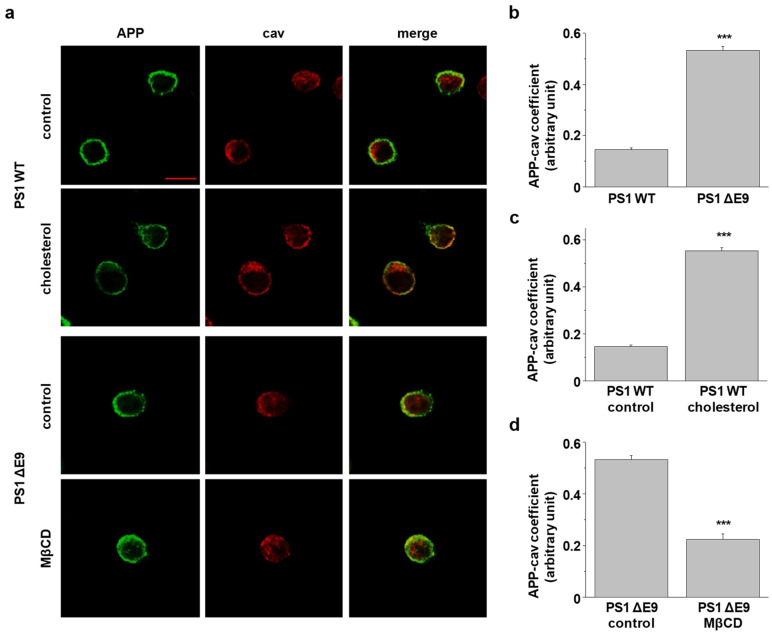
Localization of amyloid precursor protein (APP) in lipid raft microdomains was modulated by cellular cholesterol levels. Chinese hamster ovary (CHO) PS1WT cells were incubated with 150 μM β-cholesterol to increase cellular cholesterol levels. In contrast, CHO PS1 ΔE9 cells were treated with 5 mM MβCD to reduce cellular cholesterol levels. Cells were incubated with APP antibody at 4 °C to label surface APP, followed by fixing and permeabilizing. Then, cells were incubated with antibody against caveolin-1 (cav), a lipid raft marker. After washing, Alexa488- and Alexa647-conjugated secondary antibodies were used to detect primary antibodies of APP and caveolin, respectively. (**a**) Typical immunofluorescent reactivity is displayed using confocal microscopy. Data are representative of four independent experiments. Scale bars correspond to 10 μm. Colocalization of APP and caveolin were indicated between (**b**) CHO PS1 WT cells and PS1 ΔE9 (*n* = 4), (**c**) PS1 WT control and cholesterol-treated PS1 WT cells (*n* = 4), and (**d**) PS1 ΔE9 and MβCD-treated PS1 ΔE9 cells (*n* = 4). The colocalization of APP and caveolin was analyzed with Image J. Statistical analysis was performed using one-way ANOVA: *** *p* < 0.001.

We previously showed that cellular cholesterol level in Chinese hamster ovary (CHO) PS1 ΔE9 cells is upregulated compared to PS1 WT cells and that the elevated cholesterol increases APP localization in lipid rafts [38]. To investigate APP localization exclusively at the plasma membrane, we immunostained surface APP with the lipid raft marker, caveolin. CHO PS1 WT and ΔE9 cells were incubated with APP antibody (6E10) against the N-terminal region of APP at 4 °C to label APP at the plasma membrane. After fixation, cells were permeabilized and incubated with caveolin-1 antibody. Typical immunoreactivity of APP and caveolin (cav) are shown in Figure 1a. Colocalization of APP-caveolin was quantified as shown in Figure 1b–d. The coefficient was significantly higher in PS1 ΔE9 cells compared to PS1 WT cells by 3.6-fold, indicating increased localization of APP in lipid rafts (Figure 1b, *n* = 4). We manipulated membrane cholesterol levels with methyl-β-cyclodextrin (MβCD) or MβCD-cholesterol (β-cholesterol). Total cholesterol levels were measured and compared under different conditions (Supplementary Figure S1). Firstly, PS1 WT cells were treated with 150 μM β-cholesterol for 1 h. Cholesterol levels were increased by 2.2-fold by this manipulation while APP localization in caveolin-positive lipid rafts was increased by 3.8-fold (Figure 1c, *n* = 4). When PS1 ΔE9 cells were treated with 5 mM MβCD for 30 min, cholesterol levels were reduced by 27%. APP localization in caveolin-positive lipid rafts was significant decreased by this treatment (Figure 1d, *n* = 4).

Cholera toxin B (CTB) binds to ganglioside GM1, one of the components of the cholesterol-enriched lipid raft microdomains [39]. When we used CTB as another marker for lipid rafts, APP localization in lipid rafts showed similar results by the manipulation of cholesterol levels (Supplementary Figure S2). Taken together, these data suggest that increasing or decreasing cholesterol levels increases or decreases APP localization in lipid rafts, respectively.

### 2.2. Endocytosis Rate of APP Is Increased in CHO PS1 ΔE9 Cells

**Figure 2:**
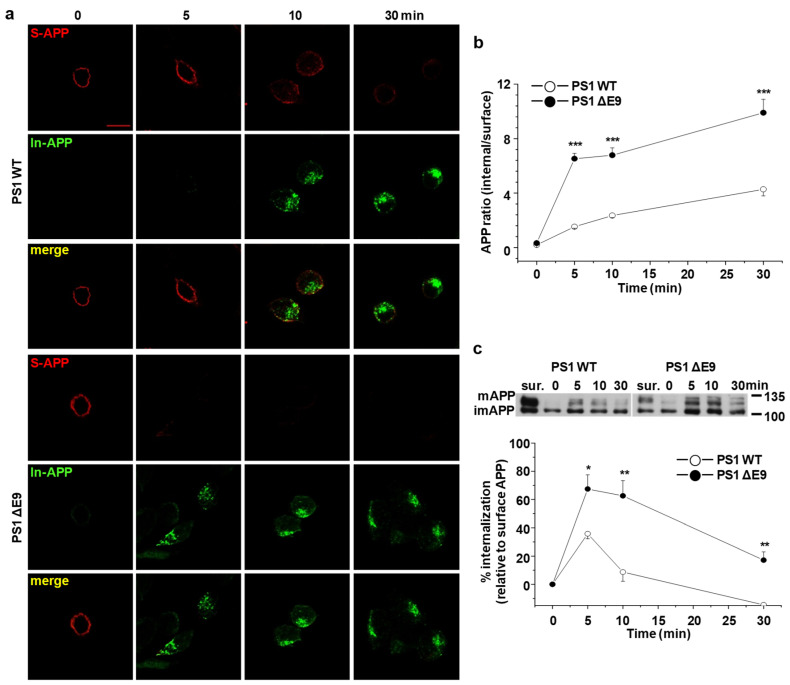
The rate of APP endocytosis was significantly increased in CHO PS1 ΔE9 cells compared to PS1 WT cells. Cells were labeled with APP antibody at 4 °C and transferred to 37 °C for indicated times to allow internalization. Then, cells were fixed and APP at cell surface was stained with Alexa647 (red)-conjugated secondary antibody (S-APP). After permeabilizing, Alexa488 (green)-conjugated secondary antibody was used to label internalized APP (In-APP). (**a**) Representative confocal image shows APP localization at each time. Data are representative of five independent experiments. Scale bars correspond to 10 μm. (**b**) Fluorescence intensities of APP were measured using Image J software. APP endocytosis was determined as the ratio of Alexa488-labeled APP/Alexa647-labeled APP (*n* = 5). (**c**) To biochemically quantify the rate of APP endocytosis, EZ-Link sulfo-NHS-SS-biotin was used as described in Methods. Only the internalized biotin-labeled proteins were isolated with streptavidin beads and the biotin labeled proteins were run on Western blot and detected with APP antibody. Total biotin-labeled APP (surface APP; sur.) is also shown. The upper panel shows a representative Western blot. The lower panel shows the rate of APP endocytosis by comparing internalized APP to surface APP (*n* = 6). Statistical analysis was performed by one-way ANOVA: * *p* < 0.05, ** *p* < 0.01, *** *p* < 0.001.

Several studies have demonstrated that cholesterol-enriched lipid rafts are associated with endocytosis of membrane-anchored proteins [40,41,42]. Since we observed that increased cholesterol levels increased the localization of APP in lipid rafts, we tested whether APP endocytosis was also affected. For this purpose, we used the primary antibody uptake method. Two different fluorescence-conjugated secondary antibodies were used to differentiate internalized APP from APP in the plasma membrane. Cells were incubated with APP antibody at 4 °C to label surface APP. After labeling, unbound-antibodies were washed and cells were incubated at 37 °C for 5, 10, and 30 min to allow for internalization of antibody-bound APP. Subsequently, cells were fixed and incubated with Alexa647-conjugated secondary antibody at 4 °C to visualize only APP remaining at the plasma membrane. After permeabilizing, internalized APP was captured by Alexa488-conjugated secondary antibody. Typical immunofluorescence reactivities for surface APP (S-APP; red) and internalized APP (In-APP; green) were shown in Figure 2a. In PS1 WT cells, internalized APP gradually increased during the time course of incubation. A significant amount of APP remained at the plasma membrane even at 10 min. In PS1 ΔE9 cells however, cell surface APP was rapidly reduced. Rates of APP endocytosis were measured by calculating the intensity ratio of internalized APP over surface APP, as shown in Figure 2b. The intensity ratio of APP at 5 min was much larger in PS1 ΔE9 cells (6.5 ± 0.4, *n* = 5) than in PS1 WT cells (1.5 ± 0.1, *n* = 5), indicating increased APP endocytosis. The increased APP endocytosis in PS1 ΔE9 cells was observed even at 30 min.

As an alternative way to quantitatively measure the rate of endocytosis, biotinylation method was used. All surface proteins were labeled with sulfo-NHS-SS-biotin at 4 °C [43,44]. After washing, cells were incubated at 37 °C for 0, 5, 10, and 30 min to allow internalization of biotin-labeled surface proteins. After initiating endocytosis, reducing agent was used to remove biotins from surface proteins. Following cell lysis, the internalized biotin-bound proteins were captured using streptavidin beads. The biotin-labeled proteins were loaded on SDS-PAGE gels. Thus, the biotin-bound APP represented internalized APP during the incubation times at 37 °C. Total surface APP levels were also measured. The representative Western blot image is shown in Figure 2c. The relative level of internalized APP was evaluated by comparing the amounts of biotin-labeled internalized APP to total surface APP (Figure 2c). At 5 min, 35.8 ± 3.1% of surface APP was internalized in PS1 WT cells, whereas 67.5 ± 1.0% of surface APP was internalized in PS1 ΔE9 cells (*n* = 6). The amounts of internalized APP were significantly higher at each time point in PS1 ΔE9 cells than in PS1 WT cells, consistent with our primary antibody uptake results. After 5 min, the amount of internalized APP diminished, which may be due to the rapid cleavage of APP by secretases once it is internalized [45]. These results suggest that the endocytosis rate of APP increased in PS1 ΔE9 cells compared to PS1 WT cells.

### 2.3. Increased Cholesterol Level Does Not Affect the Endocytosis Rate of Transferrin

It was reported that cholesterol affected both clathrin-dependent and -independent endocytosis [46,47]. To test whether the increased cholesterol level affected clathrin-dependent endocytosis, we measured transferrin endocytosis from CHO PS1 WT and ΔE9 cells. Transferrin is a well-characterized protein which is internalized via clathrin-dependent endocytosis [7]. Cells were treated with Alexa488-conjugated transferrin at 37 °C for 5, 10, and 30 min to allow its endocytosis. The residual surface transferrin was removed by acidic buffer and the remaining intracellular transferrin was visualized under fluorescent microscopy (Supplementary Figure S3). When fluorescence intensity was analyzed, the endocytosis levels of transferrin were not different between PS1 WT and ΔE9 cells. We also quantitatively measured transferrin endocytosis rate using a biochemical method. Cells were incubated with Alexa488-conjugated transferrin at 37 °C for 5, 10, and 30 min to allow its internalization. Then, the cell lysates were run on Western blotting. The fluorescent bands were detected and quantified. The endocytosis rate of transferrin in PS1 ΔE9 cells was not different from that in PS1 WT cells (Supplementary Figure S3). These observations suggest that the effect of increased cholesterol specifically increased APP endocytosis, but not clathrin-dependent endocytosis.

### 2.4. Cholesterol Levels Regulate Endocytosis Rates of APP

**Figure 3:**
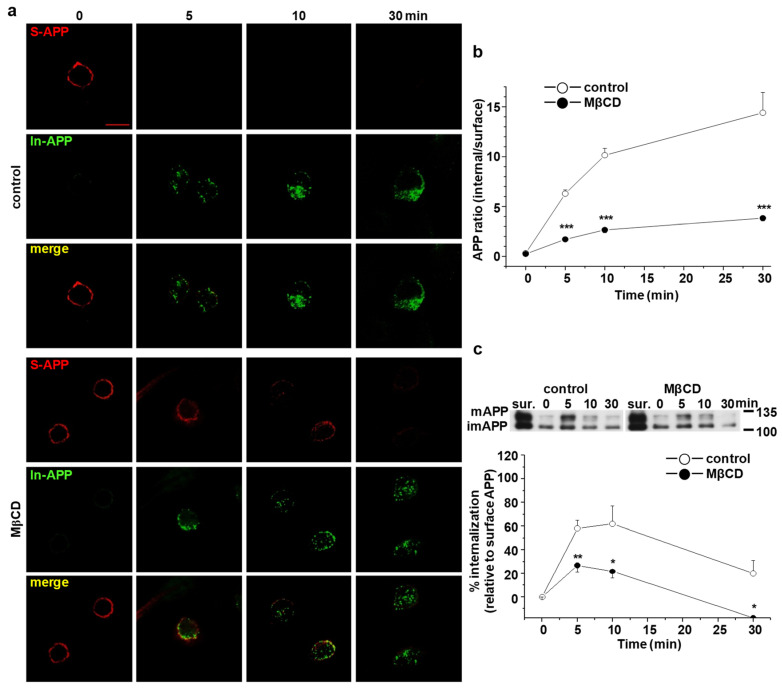
Reduction of cellular cholesterol decreased the rate of APP endocytosis in PS1 ΔE9 cells. CHO PS1 ΔE9 cells were treated with 5 mM MβCD. Cells were then labeled with APP antibody at 4 °C to monitor APP endocytosis, as described in Figure 2. (**a**) Representative confocal image shows APP localization at indicated times. Data are representative of five independent experiments. Scale bars correspond to 10 μm. (**b**) The rate of APP endocytosis was measured as the ratio of internalized APP over surface APP (*n* = 5). (**c**) APP endocytosis was quantified using EZ-Link sulfo-NHS-SS-biotin, as described in Figure 2. Total biotinylated APP (surface APP; sur.) is also shown. The upper panel shows a representative Western blot. The lower panel shows the rate of APP endocytosis by comparing internalized APP to surface APP (*n* = 5). Statistical analysis was carried out by one-way ANOVA: * *p* < 0.05, ** *p* < 0.01, *** *p* < 0.001.

To directly show that cholesterol levels affected APP endocytosis, CHO PS1 ΔE9 cells were treated with 5 mM MβCD for 30 min to reduce cellular cholesterol. Then, cells were incubated with APP antibody and further treated with two different fluorescence-conjugated secondary antibodies to visualize internalized APP and APP in the plasma membrane (Figure 3a). In MβCD-treated cells, APP remained at the plasma membrane even after 10 min internalization, which was similar to PS1 WT cells. Rates of APP endocytosis are represented by the ratio of internalized APP/surface APP (Figure 3b). At 5 min, the intensity ratio decreased from 6.3 ± 0.4 to 1.7 ± 0.1 (*n* = 5) by MβCD, indicating a significant reduction of APP endocytosis. The effect of MβCD maintained even at 30 min. Biotinylation method was also performed to measure the rate of APP endocytosis (Figure 3c). In PS1 ΔE9 control cells, APP internalization was increased gradually and peaked at 10 min. By MβCD, surface APP ratio was reduced from 58.2 ± 6.8 (*n* = 5) to 26.8 ± 5.7 (*n* = 5) at 5 min. These results indicate that decreasing cellular cholesterol levels in PS1 ΔE9 cells decreased APP endocytosis rate.

Next, we exogenously elevated cholesterol levels by treating CHO PS1 WT cells with 150 μM β-cholesterol for 1 h. The ratio of APP endocytosis was increased by 5–7 times by β-cholesterol in a time-dependent manner (Supplementary Figure S4). Thus, increasing cellular cholesterol levels in PS1 WT cells increased the rate of APP endocytosis. Taken together, these results suggest that cholesterol levels regulated APP endocytosis.

### 2.5. APP Localization in Lipid Rafts Is Regulated by Cholesterol Levels

**Figure 4:**
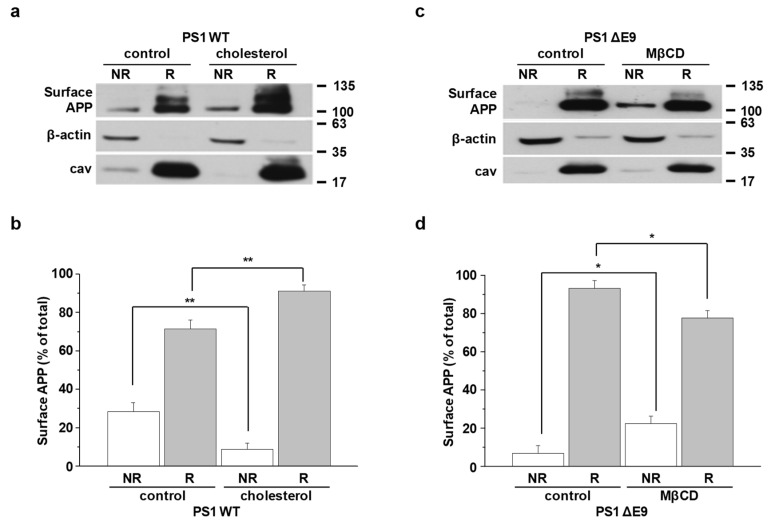
Cellular cholesterol levels determined the localization of surface APP in lipid raft microdomains. To examine APP localization at the plasma membrane, cells were incubated with EZ-Link NHS-biotin at 4 °C to label all surface proteins. Then, biotin-labeled cell lysates were pooled for discontinuous sucrose density gradient to separate lipid raft and nonlipid raft fractions. Fractions 4 to 6 (lipid rafts, R) or 8 to 12 (nonlipid rafts, NR) were combined, and the same amount of protein was used for capturing biotin-labeled proteins with streptavidin beads. The biotin labeled proteins were run on Western blot, and detected with APP, β-actin, and caveolin (lipid raft marker) antibodies. (**a**) CHO PS1 WT cells were pretreated with 150 μM β-cholesterol before biotin-labeling. A typical result demonstrates the only surface APP levels from raft and nonraft fractions (*n* = 6). (**b**) Quantitative analysis of surface APP from PS1 WT control and cholesterol-treated PS1 WT cells was shown (*n* = 6). (**c**) CHO PS1 ΔE9 cells were pretreated with 5 mM MβCD to decrease cholesterol levels before labeling surface proteins. Representative Western blot result indicates surface APP levels from raft and nonraft fractions (*n* = 5). (**d**) Quantitative analysis of surface APP from PS1 ΔE9 control and MβCD-treated PS1 ΔE9 cells was shown (*n* = 5). Statistical analysis was carried out by one-way ANOVA: * *p* < 0.05, ** *p* < 0.01.

Although confocal microscopy results in Figure 1 supported that APP is associated in lipid rafts, it is still inconclusive because of the insufficient resolution for nanoscale lipid raft microdomains. To overcome this limitation and quantitatively measure only surface APP localization in lipid rafts, we designed a new, biotin-labeled lipid raft fractionation method. Cells were pretreated with EZ-Link NHS-biotin at 4 °C to label cell surface proteins. After unbound biotins were washed, cells were harvested, homogenized, and sonicated. Equal amounts of biotin-bound proteins from cell lysates were loaded on discontinuous sucrose density gradients, as described in Methods. Equal volumes from 12 recovered gradient fractions were run on Western blot to detect APP, β-actin (a nonlipid raft marker), and caveolin (a lipid raft marker), as shown for a typical result in Supplementary Figure S5. When levels of cholesterol and protein were measured, cholesterol-enriched lipid raft fractions (4 to 6) were clearly separated from protein-enriched nonraft fractions (8 to 12). Protein levels in each fraction did not differ between the PS1 WT and ΔE9 cells. However, cholesterol levels in lipid raft fractions were significantly higher in PS1 ΔE9 cells than in PS1 WT cells, as we reported previously [38]. For better quantification of APP levels in lipid rafts and nonrafts, we combined fractions from 4 to 6 (lipid raft fractions, R) and fractions from 8 to 12 (nonraft fractions, NR). Equal amounts of biotin-bound surface proteins were captured with streptavidin beads. Then, equal volumes of each fraction were loaded on gels for Western blots. Thus, the biotin-bound APP represented the only cell surface APP localized either in lipid rafts or in nonrafts. A typical result is shown in Figure 4a,c. Higher APP level in lipid rafts than in nonrafts was due to significantly lower total protein levels in lipid rafts than in nonrafts. Thus, APP comprised a larger proportion of the total proteins in lipid rafts. The ratio of surface APP levels in each fraction was calculated as shown in Figure 4b,d. In PS1 ΔE9 control cells, most surface APP was localized in lipid rafts and barely detectable in nonlipid rafts (Figure 4c). APP level in raft fractions was higher in PS1 ΔE9 cells (93.2 ± 4.0%, *n* = 5) than in PS1 WT cells (71.6 ± 4.6%, *n* = 6), which was consistent with our previous co-staining result in Figure 1. Conversely, surface APP level in nonraft fractions was lower in PS1 ΔE9 cells compared to PS1 WT cells.

To investigate the effect of cholesterol levels on surface APP localization, PS1 WT cells were pretreated with 150 μM β-cholesterol for 1 h (Figure 4a) and PS1 ΔE9 cells were pretreated with 5 mM MβCD for 30 min (Figure 4c). APP levels in lipid rafts were significantly increased to 91.3 ± 3.3% (*n* = 6) by increasing cholesterol levels (Figure 4b). Conversely, surface APP localization in nonrafts was significantly decreased by β-cholesterol. Reducing cholesterol level increased the localization of surface APP in nonlipid rafts. The ratio of APP in nonlipid raft fractions increased from 6.8 ± 4.0% in control cells to 22.3 ± 3.9% (*n* = 5) in MβCD-treated cells (Figure 4d). In contrast, the ratio of APP in lipid rafts showed a significant decrease from 93.2 ± 4.0% to 77.7 ± 3.9% (*n* = 5) by MβCD. These results indicate that modulating cholesterol levels regulated surface APP localization in lipid rafts, which could affect the further processing of APP and subsequent Aβ production.

### 2.6. APP Preferentially Internalizes from Lipid Rafts

**Figure 5:**
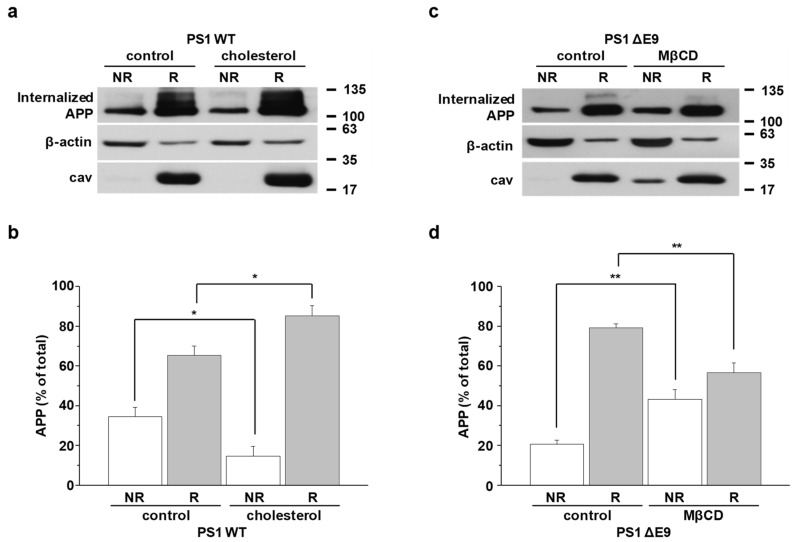
Cellular cholesterol levels determined raft-dependent endocytosis of APP. To verify the contribution of lipid rafts to APP endocytosis, cells were incubated with EZ-Link NHS-SS-biotin at 4 °C to label surface proteins. Then, cells were incubated at 37 °C for 10 min to allow internalization of biotin-labeled surface proteins. After internalization, the remaining surface-bound biotin was removed and biotin-labeled cell lysates were loaded for discontinuous sucrose density gradient centrifugation to separate lipid raft and nonlipid raft fractions. Fractions 4–6 (lipid rafts, R) or 8–12 (nonlipid rafts, NR) were pooled, and biotin-labeled proteins were captured with streptavidin beads. Internalized biotin-labeled proteins were loaded on Western blots and detected with APP, β-actin, and caveolin (lipid raft marker) antibodies. (**a**) CHO PS1 WT cells were pretreated with 150 μM β-cholesterol before labeling surface proteins. Internalized biotin-bound APP levels from raft and nonraft fractions were detected by Western blotting (*n* = 5). (**b**) Analysis of band densitometry shows the levels of internalized labeled APP from PS1 WT control and cholesterol-treated PS1 WT cells (*n* = 5). (**c**) CHO PS1 ΔE9 cells were pretreated with 5 mM MβCD to reduce cholesterol levels before biotin-labeling. Internalized labeled APP from lipid raft and nonlipid raft fractions were monitored by Western blot (*n* = 6). (**d**) The band densitometry of internalized labeled APP from lipid raft and nonlipid raft fractions was analyzed from PS1 ΔE9 control and MβCD-treated PS1 ΔE9 cells (*n* = 6). Statistical analysis was carried out by one-way ANOVA: * *p* < 0.05, ** *p* < 0.01.

Our findings showed that cholesterol levels regulated the localization of surface APP in lipid rafts as well as APP endocytosis in general. However, it is still unclear whether lipid raft microdomains are the sites where APP undergoes endocytosis. To investigate this possibility, we firstly applied a reversible biotinylation method to lipid rafts fractionation. Cells were pretreated with EZ-Link NHS-SS-biotin to label surface proteins, and then further incubated at 37 °C for 10 min to allow internalization of biotin-labeled surface proteins as described in Methods. All remaining surface biotins were then removed using reducing agents. Equal amounts of biotin-bound proteins from cell lysates were loaded on discontinuous sucrose density gradient to obtain 12 fractions. The equal amount of proteins from lipid raft fractions (R; fractions 4 to 6) and nonraft fractions (NR; fractions 8 to 12) were captured with streptavidin beads to pull down biotin-bound proteins. Then, the equal volumes of lipid rafts and nonlipid rafts were loaded for Western blots. If the characteristics of the internalized membranes from lipid raft and nonraft fractions are maintained, the bead-captured APP represents the internalized APP originating from either lipid rafts or nonrafts. A typical result is shown in Figure 5a,c. The ratios of internalized APP from lipid rafts and nonrafts were calculated and shown in Figure 5b,d. The ratio of APP internalized from lipid raft fractions was higher in PS1 ΔE9 cells (79.3 ± 2.1%, *n* = 6) than in PS1 WT cells (65.5 ± 4.7%, *n* = 5). Considering that surface APP level in raft fractions was higher in PS1 ΔE9 cells than in PS1 WT cells (Figure 4), this result may suggest that APP is preferentially internalized from lipid rafts.

Next, we investigated the effects of cholesterol on APP internalization from lipid rafts. As shown for a typical result in Figure 5a, the ratio of internalized APP from lipid raft fractions was increased to 85.4 ± 5.1% (Figure 5b, *n* = 5) by β-cholesterol. In contrast, when we decreased cholesterol levels from PS1 ΔE9 cells, the ratio of internalized APP from raft fractions decreased to 56.7 ± 4.9% (Figure 5d, *n* = 6). Thus, increasing or decreasing cholesterol levels consistently induced the same changes in the ratio of internalized APP from lipid rafts. These results suggest that cholesterol levels affected APP endocytosis from lipid rafts by regulating APP localization in these specific microdomains.

Lipid raft microdomains are heterogeneous and very dynamic structures with various physiological–chemical properties dependent on cell types [26]. To test whether cholesterol effects on APP localization in lipid raft were cell-type-dependent, we used HeLa cells stably transfected with APP751 carrying the Swedish mutation (APPswe). Cells were incubated with 150 μM β-cholesterol for 1 h or 1 mM MβCD for 30 min to modulate cellular cholesterol levels. Then, the biotin-labeled lipid raft fractions were obtained to monitor the membrane localization of surface APP (Supplementary Figure S6) and raft-dependent APP endocytosis (Supplementary Figure S7). We obtained similar results in HeLa cells as in CHO cells. From these results, we confirmed that the effects of cholesterol on APP localization in lipid rafts as well as on APP endocytosis from lipid rafts were not cell-type-dependent.

### 2.7. Localization of Endogenous Neuronal APP in Lipid Rafts at the Plasma Membrane Is Determined by Cellular Cholesterol Levels

**Figure 6:**
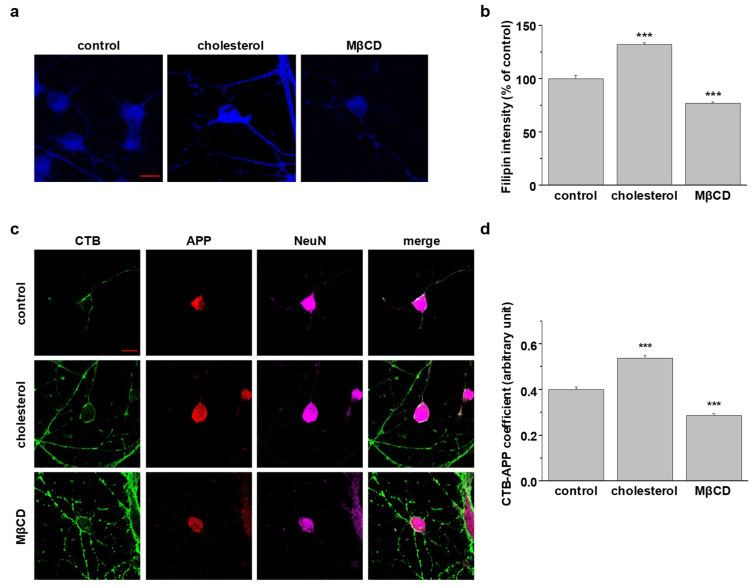
Cellular cholesterol levels determined the localization of endogenous APP in lipid raft microdomains from a rat’s primary hippocampal neurons. (**a**) Rats’ primary hippocampal neurons (DIV21-DIV23) were incubated with either 1.5 mM β-cholesterol or 2 mM MβCD. Then, cells were incubated with filipin to stain free cholesterol levels. Representative confocal images are shown. Data are representative of three independent experiments. Scale bars correspond to 10 μm. (**b**) Filipin fluorescent intensities was analyzed with Image J (*n* = 3). (**c**) Hippocampal neurons were treated with β-cholesterol or MβCD, followed by incubation with APP antibody and cholera toxin B (CTB) at 4 °C. After fixing and permeabilization, neurons were incubated with the NeuN antibody to detect neurons. Confocal image from four independent experiments is shown. Scale bars correspond to 10 μm. (**d**) Coefficiency of APP and CTB was analyzed with Image J (*n* = 4). Statistical analysis was performed by one-way ANOVA: *** *p* < 0.001.

To test whether cholesterol levels in neurons affect the localization of surface APP in lipid rafts, we used primary hippocampal neurons from Sprague-Dawley rat embryos. Previous research showed that these neurons are fully matured at 21–35 days in vitro (DIV21-DIV35; stationary phase) and are characterized by pyramidal cell bodies, intensive connections, and networking of neuritis [48]. Neurons (DIV21-DIV23) were incubated with 2 mM MβCD or 1.5 mM β-cholesterol for 30 min. Then, neurons were incubated with 300 μg/mL filipin for 2 h to stain free cholesterol, as shown in Figure 6a. β-Cholesterol treatment increased the filipin intensity by 32.0 ± 3.3% (*n* = 3) while MβCD treatment decreased it by 23.0 ± 1.2% (*n* = 3) (Figure 6b). To monitor the localization of APP at the plasma membrane, neurons were costained with APP antibody and CTB, a lipid raft marker. After fixing and permeabilizing, NeuN antibody was used to identify neurons. Both the cell bodies and axons were stained with CTB (Figure 6c). The endogenous neuronal APP was mostly localized in the cell bodies. Elevated cholesterol increased the colocalization of APP-CTB, while MβCD decreased the colocalization of APP-CTB (Figure 6d, *n* = 4). These results suggest that lipid rafts localization of endogenous surface APP was regulated by cholesterol levels, consistent with the results obtained from APP-transfected cells.

### 2.8. Disruption of Lipid Raft Microdomains Attenuates APP Endocytosis in SH-SY5Y-APP/BACE1 Cells

**Figure 7:**
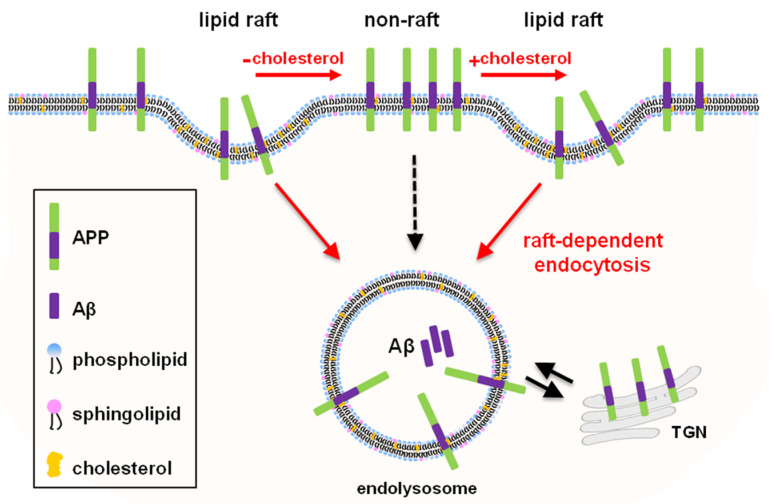
Current model for our study.

**Figure 8:**
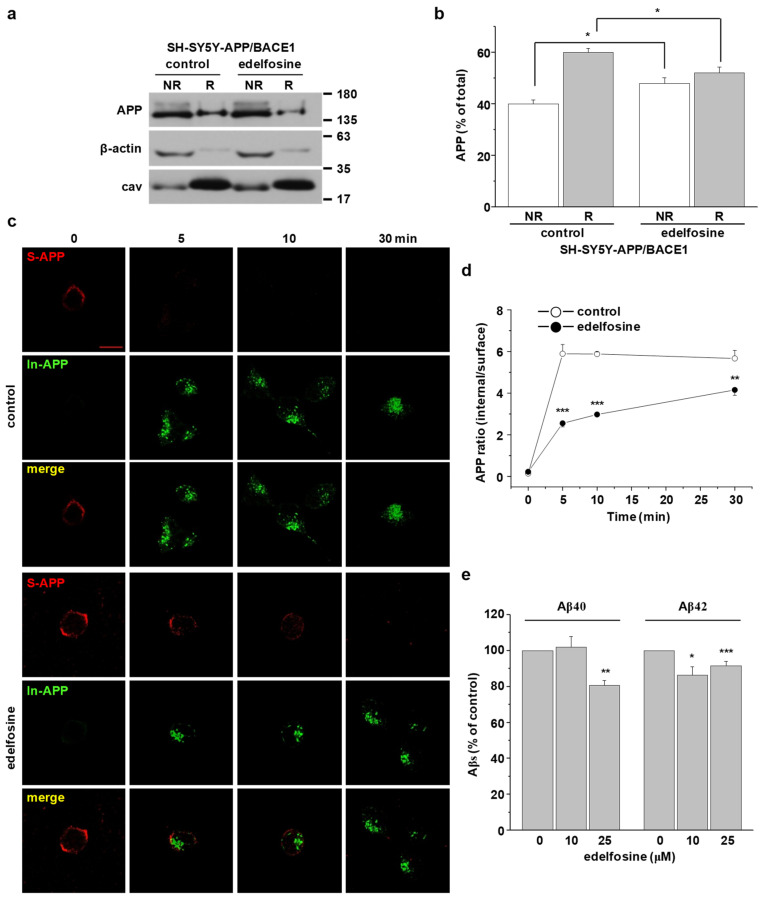
Disruption of lipid rafts with edelfosine-attenuated raft-dependent endocytosis of APP and decreased Aβ42 levels in SH-SY5Y-APP/BACE1 cells. (**a**) SH-SY5Y-APP/BACE1 cells were pretreated with 10 μM edelfosine. A typical result demonstrated APP localization from raft and nonraft fractions (*n* = 5). (**b**) Quantitative analysis of APP from control group and edelfosine-treated group (*n* = 5). (**c**) SH-SY5Y-APP/BACE1 cells were incubated with 10 μM edelfosine to disturb lipid rafts. Using the primary antibody uptake method with two different fluorescent-conjugated secondary antibodies as described in Figure 2, surface APP and internalized APP were labeled separately. Confocal image is representative of three independent experiments. Scale bars correspond to 10 μm. (**d**) APP endocytosis rate was calculated by the ratio of internalized APP to surface APP (*n* = 3). (**e**) SH-SY5Y-APP/BACE1 cells were treated with 0, 10, or 25 μM of edelfosine, and then the levels of Aβ40 and the levels of Aβ42 were measured from the conditioned media (*n* = 5). Statistical analysis was carried out using one-way ANOVA: * *p* < 0.05, ** *p* < 0.01, *** *p* < 0.001.

Our findings are summarized as a model in Figure 7, suggesting the direct relationship between APP localization in lipid rafts and raft-dependent APP endocytosis, which leads to Aβ production. To prove this concept, we used edelfosine (1-*O*-octadecyl-2-*O*-methylrac-glycero-3-phosphocholine), an alkyl-lysophospholipid (ALP). Edelfosine is used as an anticancer drug [49,50]. According to molecular dynamic simulation, X-ray diffraction, and ^2^H nuclear magnetic resonance, it has been reported that edelfosine disorganizes lipid membranes by accumulating into cholesterol-rich lipid rafts since it shows high affinity for cholesterol [49,50,51,52]. It also interferes with lipid-raft-based signal transductions [49,53,54,55,56,57,58]. These previous findings suggest that edelfosine may disrupt lipid raft microdomains. According to our model, disruption of lipid rafts would affect raft-dependent APP endocytosis and Aβ production. We used human neuroblastoma SH-SY5Y cells stably transfected with APP751 and BACE1 (SH-SY5Y-APP/BACE1). Normal SH-SY5Y cells showed very low levels of endogenous APP [38]. Cells were incubated with 10 μM edelfosine for 2 h, then lipid raft fractionation was performed. As shown in Figure 8a, edelfosine significantly decreased the ratio of APP in lipid rafts from 60.0 ± 1.6% to 52.1 ± 2.3% (Figure 8b, *n* = 5). In contrary, APP ratio in nonrafts was significantly increased from 40.0 ± 1.6% to 47.9 ± 2.3% by edelfosine (Figure 8b, *n* = 5). Next, APP endocytosis was monitored with primary antibody uptake method with two different fluorescent-conjugated secondary antibodies. Typical immunoreactivities of surface APP and internalized APP were shown in Figure 8c. Surface APP level was dramatically reduced at 5 min in control cells, whereas a significant amount of APP remained at the cell surface even at 10 min in edelfosine-treated cells. The rate of APP endocytosis was analyzed by the ratio of internalized APP/surface APP as shown in Figure 8d. The intensity ratio of APP at 5 min was decreased from 5.9 ± 0.5 to 2.6 ± 0.2 by edelfosine (*n* = 3). The effect of edelfosine on APP endocytosis was also evident at 10 min, and maintained at 30 min. These results suggest that disruption of cholesterol-enriched rafts by edelfosine redistributed APP into nonlipid rafts and also attenuated APP internalization.

We then measured secreted Aβ40 and Aβ42 levels from the conditioned media after SH-SY5Y-APP/BACE1 cells were incubated with 0, 10, or 25 μM edelfosine for 4 h. With a concomitant decrease in the generation of Aβ40, edelfosine decreased secreted Aβ42 levels in a dose-dependent manner (Figure 8e, *n* = 5). At 25 μM, edelfosine decreased Aβ42 by 19.2%. Consistent with our model in Figure 7, these results suggest that displacing APP from lipid rafts by disruption of lipid rafts reduced APP endocytosis, resulting in decreased Aβ production.

## 3. Discussion

Many lines of evidence shed light on the role of cholesterol in AD [13,59]. Changes in cholesterol homeostasis as well as other lipid classes in the postmortem AD brain are considered to represent a third pathological feature of AD following the extracellular senile plaques and the intracellular neurofibrillary tangles [19,20,22,23]. Recently, alteration of cholesterol biosynthesis was found at a very early stage of AD model mice [60]. APP processing is highly influenced by membrane cholesterol since APP and the associated secretases are localized in lipid raft structures [28,30,31,61,62]. Cholesterol-enriched lipid rafts have also been implicated in the regulation of several membrane trafficking and cell signaling pathways [40,41,42]. These findings suggest that cellular cholesterol levels and the distribution of APP in lipid rafts are critical for APP processing and Aβ generation. Indeed, we found that Aβ42 production was closely related to cholesterol levels (Supplementary Figure S8). However, the precise role of cholesterol-enriched lipid raft microdomains on APP processing and Aβ production remains unclear.

Based on the colocalization evidence, elevated cholesterol levels in PS1 ΔE9 cells increased APP localization in both caveolin- and CTB-positive regions, which represent raft microdomains. Manipulation of cellular cholesterol with MβCD or β-cholesterol decreased or increased the degree of colocalization of APP-caveolin and APP-CTB, respectively. In addition to APP-expressing cells, we also confirmed the effects of cellular cholesterol on the localization of endogenous APP in raft microdomains in primary hippocampal neurons. These findings demonstrate that surface APP localization in lipid rafts is regulated by cellular cholesterol levels. The increased localization of APP in lipid rafts by the elevated cholesterol supports previous reports showing that cholesterol binds to the transmembrane region of APP/C99 and favors the formation of APP/C99-cholesterol complex [63,64,65,66,67].

Endocytosis is a serial process of membrane dynamics. Therefore, membrane lipid composition is critical for endocytic mechanisms [7,8,9]. Additionally, the distribution of membrane proteins between lipid rafts and nonlipid rafts is thought to regulate the endocytosis of certain proteins [7,8]. Consistent with these results, we found that endogenously elevated cholesterol in PS1 ΔE9 cells caused increased APP endocytosis compared to PS1 WT cells. However, the endocytosis of transferrin was not changed by the altered cholesterol levels. These results may suggest that the elevated cholesterol levels specifically affect internalization of APP, while general clathrin-dependent endocytosis is not affected. To prove whether lipid raft microdomains are the site for APP endocytosis, we designed a new experiment—biotin-labeled lipid raft fractionation. With this biochemical technique, we not only provided new evidence for the contribution of lipid raft microdomains to APP endocytosis, but also quantitatively measured the internalized APP derived from both rafts and nonrafts. We found that the internalized APP was derived from both lipid rafts and nonlipid rafts. Intriguingly, the elevated cholesterol level in PS1 ΔE9 cells induced preferential internalization of APP from lipid rafts but not from nonrafts. When we partially depleted cholesterol levels with MβCD, the portion of internalized APP derived from lipid rafts decreased. Thus, the results from up-/down-regulation of cholesterol levels indicated that membrane rafts are critical for raft-dependent APP internalization. In addition, these cholesterol effects on APP localization and raft-dependent APP endocytosis were not cell-type-specific since we recapitulated these effects using HeLa APPswe cells. Taken together, the current study suggests the novel possibility that exogenously added cholesterol increases surface APP localization in lipid rafts and raft-dependent APP endocytosis. However, the exogenously added cholesterol might also alter the distribution of β- and γ-secretases in lipid raft microdomains [28,29,30,31].

At the plasma membrane, APP is either cleaved by α-secretase or undergoes endocytosis through clathrin-dependent/-independent endocytosis within minutes. Since we confirmed that raft-dependent APP endocytosis is sensitive to cellular cholesterol levels, we also determined the subcellular localization of APP following its internalization (Supplementary Figure S9). We found that modification of cholesterol level affected the amount of internalized APP from lipid rafts reaching early endosomes. However, the rate of internalization was not affected by cholesterol levels. 

According to Cossec and colleagues, APP endocytosis is increased by cholesterol in a clathrin-dependent manner [37]. Considering our result, showing that internalized APP originated from lipid rafts is sensitive to cholesterol modulation, there may exist different pathways of APP endocytosis besides clathrin-mediated APP internalization. It has been proposed that the lipid-raft-associated protein, flotillin, can cluster with APP into lipid rafts by binding to its C-terminal region [68,69]. APP can also bind cholesterol through its transmembrane domain [62,63,64,65,66]. Taken together, it is possible that APP associated with either cholesterol or flotillin is recruited to raft microdomains, and that recruited APP in lipid rafts may be internalized to the endosomal/lysosomal subcellular trafficking system. This form of APP endocytosis may occur via raft-dependent pathways such as caveolin-/flotillin-mediated endocytosis [40,41,42]. Clathrin-dependent APP endocytosis is also affected by cellular cholesterol [37,46,47], which is consistent with our result showing that the internalized APP from nonrafts was also changed by alterations of cholesterol levels. Considering the previous reports, our results may suggest that APP can be internalized through multiple pathways including both classical clathrin-dependent endocytosis and raft-induced endocytosis. Further studies would be needed to test this possibility.

The localization of APP in lipid rafts is closely related to APP processing and Aβ production, as shown in our model (Figure 7). We used edelfosine to prove this relationship. Edelfosine removed APP from lipid raft fractions probably due to its high affinity for cholesterol [49,52]. As we predicted from the model, edelfosine also attenuated the APP endocytosis rate, decreasing Aβ40 and Aβ42 levels. Thus, our results may suggest that edelfosine and other ALPs could be a new class of AD treatment targeting lipid rafts. However, further studies are needed, including on the in vivo effects of ALPs to reduce Aβs.

In summary, cholesterol recruited surface APP into lipid raft microdomains. Surface APP in cholesterol-enriched raft microdomains is closely related to the amyloidogenic processing of APP by increasing raft-dependent APP endocytosis. Our results demonstrate that regulating the lateral accumulation of APP in lipid rafts could be a promising and effective new therapeutic target for AD.

## 4. Materials and Methods

### 4.1. Cell Culture and Experimental Treatments

Wild-type human APP751 expressing Chinese hamster ovary (CHO) cells [70] were stably transfected with either presenilin 1 wild type (PS1 WT) or ΔE9 mutant (PS1 ΔE9). Stable CHO PS1 WT and ΔE9 cell lines were grown in Dulbecco’s Modified Eagle Medium (DMEM) supplemented with 10% (*v/v*) heat-inactivated fetal bovine serum (FBS), 100 U/mL penicillin, 100 μg/mL streptomycin, and 250 μg/mL Zeocin at 37 °C with 5% CO_2_ atmosphere. For cholesterol modulation, CHO PS1 WT cells were incubated with 150 μM MβCD-cholesterol (β-cholesterol; cholesterol-water soluble; Sigma, St. Louis, MO, USA, #C4951) for 1 h and CHO PS1 ΔE9 cells were treated with 5 mM methyl-β-cyclodextrin (MβCD; Sigma, #332615) for 30 min.

HeLa cells stably expressing APP751 carrying the Swedish mutation (APPswe) were maintained in DMEM with 10% heat-inactivated FBS, 100 units/mL penicillin, 100 μg/mL streptomycin, 250 μg/mL Zeocin, and 400 μg/mL G418 at 37 °C with 5% CO_2_ atmosphere. HeLa APPswe cells were incubated with either 1 mM MβCD or 150 μM β-cholesterol for 30 min to reduce or increase cellular cholesterol levels, respectively.

Human neuroblastoma SH-SY5Y cells stably expressing wild-type human APP and wild-type BACE (SH-SY5Y-APP/BACE1) were used. Cells were maintained in Dulbecco’s Modified Eagle Medium (DMEM) supplemented with 10% (*v/v*) heat-inactivated fetal bovine serum (FBS), 100 U/mL penicillin, 100 μg/mL streptomycin, and 250 μg/mL Zeocin at 37 °C in an atmosphere containing 5% CO2. SH-SY5Y-APP/BACE cells were treated with 10 or 25 μM edelfosine (Tocris, Bristol, Britain #3022) for 30 min to disrupt lipid raft microdomains.

### 4.2. Rat Primary Hippocampal Neuron Culture

Embryonic 18-day-old Sprague-Dawley rat fetuses were prepared for rat primary hippocampal neurons. All procedures were carried out in accordance with the guidelines of Sungkyunkwan University Animal Care and Ethics Committee. Rat primary hippocampus from both hemispheres were dissected and rinsed with Hank’s balanced salt solution (HBSS). Then, hippocampi were incubated with HBSS solution containing 0.25% trypsin-EDTA at 37 °C for 5 min. Next, the tissue was incubated with 1 mL FBS in 4 mL HBSS at 37 °C for 3 min to inactivate trypsin-EDTA and then washed three times with HBSS. Next, tissue was resuspended in neurobasal medium and filtered through a 100-μm strainer to dissect tissue into single neurons. Then, neurons were washed with HBSS and resuspended in neurobasal medium supplemented with 2% B27, 2 mM glutamax, and 1% penicillin/streptomycin (all supplements from GIBCO). Finally, primary hippocampal neurons were grown on poly-D-lysine coated glass cover slips, and neurons were maintained for 21 to 23 days in vitro (DIV21-DIV23). For cholesterol manipulation, neurons were treated with either 1.5 mM β-cholesterol or 2 mM MβCD for 30 min to increase or decrease cholesterol, respectively.

### 4.3. Filipin Staining

Filipin (Sigma-Aldrich, St. Louis, MO, USA, #F9765) staining was performed to monitor the levels of free cholesterol from rat primary hippocampal neurons. Neurons were placed on poly-D-lysine-coated cover slips and were treated with either 1.5 mM β-cholesterol or 2 mM MβCD for 30 min to increase or decrease cholesterol, respectively. Then, neurons were fixed with 4% paraformaldehyde in 4% sucrose for 15 min at room temperature. Neurons were washed and incubated with 1.5 mg/mL glycine in HBSS for 10 min at room temperature to quench the paraformaldehyde. In the dark, neurons were stained with 300 μg/mL filipin in HBSS for 2 h at room temperature. The fluorescence intensity was observed by confocal microscopy using a model microscope (LSM710, Zeiss, Göttingen, Germany). The fluorescence intensity in the region of interest according to the cell shape was measured using ImageJ program (version 1.52v).

### 4.4. Colocalization Experiments

Cells were grown on poly-D-lysine coated cover glass. After washing with ice-cold phosphate-buffered saline (PBS), cells were incubated with APP antibody (6E10; BioLegend, San Diego, CA, USA, monoclonal, #803002) at 4 °C for 1 h to label cell surface APP. Then, cells were fixed with 4% paraformaldehyde in 4% sucrose for 15 min and permeabilized in PBS containing 0.1% Triton X-100 and 2% bovine serum albumin (BSA) for 5 min. After washing, cells were blocked with 1% BSA in PBS solution for 1 h and then incubated with caveolin antibody (cav-1; BD Transduction Laboratories, San Jose, CA, USA, polyclonal, #610059) for 2 h in blocking buffer to detect lipid raft microdomains. Following washing with PBS, cells were incubated with goat antimouse conjugated with Alexa Fluor 488 (Invitrogen, Carlsbad, CA, USA, #A11001) and donkey antirabbit conjugated with Alexa Fluor 647 (Invitrogen, #A31573) secondary antibodies in blocking buffer at 4 °C for 16 h to label primary antibodies APP and cav-1, respectively. The next day, cells were washed with PBS and mounted with mounting medium (Sigma, #F6182). Immunofluorescence staining was monitored on a confocal microscope (LSM710, Zeiss).

For colocalization with cholera toxin B (CTB), CHO cells were incubated with 6E10 and 10 μg/mL Fluorescein isothiocyanate (FITC)-conjugated cholera toxin B subunits (Sigma, #C1655) to label lipid raft microdomains at 4 °C for 1 h before fixation. Then, cells were processed following the steps described above. APP was captured with goat antimouse conjugated with Alexa 647 (Invitrogen, #A21240) secondary antibody.

Primary hippocampal neurons were grown on poly-D-lysine cover slips and stained with 6E10 to label endogenous APP at the plasma membrane and 7 μg/mL FITC-conjugated CTB subunits to label lipid raft microdomains at 4 °C for 1 h. Then, neurons were fixed with 4% paraformaldehyde in 4% sucrose for 15 min and permeabilized in PBS containing 0.1% Triton X-100/2% BSA for 5 min. After 1 h blocking with 2% BSA in PBS, neurons were incubated with α-NeuN antibody (Millipore, Burlington, VT, USA, polyclonal, #ABN78) for 2 h at room temperature to detect neurons. Following washes with PBS, cells were incubated with goat antimouse conjugated with Alexa Fluor 568 (Invitrogen, #A11004) and donkey antirabbit conjugated with Alexa Fluor 647 in blocking buffer overnight to detect primary antibodies 6E10 and α-NeuN, respectively. The next day, cells were washed and mounted with mounting medium. Immunofluorescence reactivity was captured on a confocal microscope (LSM710, Zeiss). The colocalization of APP and caveolin or APP and CTB was calculated using the JACop plug-in of Image J program (https://imagej.net/Colocalization_Analysis), which is based on Mander’s correlation coefficient [71].

### 4.5. Primary Antibody Uptake Assay

CHO PS1 WT and ΔE9 cells were grown on glass cover slips coated with poly-D-lysine. The next day, cells were washed three times with ice-cold PBS for 5 min and incubated with 6E10 antibody (1:100) in PBS containing 2% BSA for 1 h at 4 °C to label surface APP at the plasma membrane. Next, cells were washed with ice-cold PBS and transferred to 37 °C for various times to allow internalization. At 0 min, cells were fixed with 4% paraformaldehyde in 4% sucrose for 15 min, followed by incubation with goat antimouse Alexa647 (red)-conjugated secondary antibody at 4 °C for 1 h to label surface APP. After internalization, cells were fixed and incubated with goat antimouse Alexa647 (red)-conjugated secondary antibody at 4 °C for 1 h to label surface APP. Next, cells were washed with PBS and then permeabilized in PBS containing 0.1% Triton X-100 and 2% BSA for 5 min. Cells were blocked with 1% BSA in PBS for 1 h at room temperature. Cells were washed with PBS twice and incubated with goat antimouse Alexa488 (green)-conjugated secondary antibody in blocking buffer for 16 h to label internalized APP. The following day, cells were washed with PBS, mounted with mounting medium, and left overnight at 4 °C to dry. Immunofluorescence reactivity was captured with a confocal microscope (LSM710, Zeiss). Mean fluorescence intensity values corresponding to plasma membrane as measured from the edge of the cell to a depth of 500 nm (surface APP; A) and mean fluorescence intensity values from the region of interest (ROI) according to the cell shape (internalized APP; B) were measured using the Image J program. The rate of APP endocytosis was obtained by calculating the ratio of B/A.

### 4.6. Transferrin Uptake

Cells were incubated with 25 μg/mL Alexa-Fluor-488-conjugated transferrin (Invitrogen, #T-13342) in PBS containing 0.1% BSA for 5, 10, and 30 min at 37 °C. Surface transferrin was removed from the plasma membrane by treating cells with pH 5.5 buffer (0.1 M sodium acetate, 0.05 M NaCl) for 5 min. Cells were then washed with PBS and fixed with 4% paraformaldehyde in 4% sucrose for 15 min at room temperature. Immunofluorescence reactivity was captured using a confocal microscope (LSM710, Zeiss). The Image J program was used to measure mean fluorescence intensity values from the region of interest (ROI) according to the cell shape.

To quantitatively measure fluorescence intensity of transferrin internalization, cells were incubated in PBS with 0.1% BSA containing 25 μg/mL Alexa-Fluor-488-conjugated transferrin for 5, 10, and 30 min at 37 °C. Then, cells were washed with PBS and lysed with lysis buffer (50 mM Hepes. pH 7.2, 100 mM NaCl, 1% Triton X-100, 1 mM sodium orthovanadate, and protease inhibitor mixture). The same amount of cell lysate protein was loaded on Tris-glycine SDS-PAGE gel. Proteins were transferred to nitrocellulose membrane, and the intensity of the bands was detected using LAS 3000 (Fuji Film, Tokyo, Japan). Band densitometry was analyzed using Multi Gauge V3.0 program (version 3.0).

### 4.7. Reversible Biotinylation Assay

CHO cells were washed with ice-cold PBS and incubated in PBS supplemented with 0.25 mg/mL sulfo-NHS-SS-biotin (Thermo, Waltham, MA, USA, #21441) for 10 min at 4 °C. Excess biotin was washed out with ice-cold PBS containing 100 mM glycine. Cells were incubated with 1% BSA in PBS for 15 min at 4 °C. After washing with PBS, cells were incubated at 37 °C for the appropriate times. For the zero-minute time point, cells were kept at 4 °C as controls. Cells were quickly washed in ice-cold PBS to stop internalization. Remaining cell surface biotin was cleaved off by incubating twice with reducing agent (50 mM sodium-2-mercapoethanesulfomate, 150 mM NaCl, 1 mM EDTA, 0.2% BSA, 20 mM Tris HCl, pH 8.6) for 25 min at 4 °C. This reaction was quenched by ice-cold PBS containing 5 mg/mL iodoacetamide (Sigma, #I1149) with 1% BSA for 10 min. We also incubated cells in PBS supplemented with 0.25 mg/mL sulfo-NHS-biotin (Thermo, #21217) at 4 °C to detect all surface proteins. For total pool of surface APP, this sample does not undergo the above reducing and quenching steps. After washing, cells were extracted in lysis buffer (50 mM Hepes, pH 7.2, 100 mM NaCl, 1% Triton X-100, 1 mM sodium orthovanadate, and protease inhibitor mixture). Equal amounts of proteins were incubated with streptavidin-agarose slurry (Millipore, #16-126) at 4 °C for 16 h in order to pull down all biotin-labeled proteins. After washing, the bound material was analyzed by Western blot. We calculated the rate of APP internalization as described below.

A: the levels of total surface APP (sulfo-NHS-biotin incubated, nonreduced, and nonquenched).
B: control (0 min; sulfo-NHS-SS-biotin incubated, kept at 4 °C).
C: internalized APP at 37 °C (sulfo-NHS-SS-biotin incubated and internalized 5, 10, or 30 min)
The (C-B)/A ratio represented the rate of internalized APP during each time point.

### 4.8. Western Blotting

The proteins were loaded on 8–10% Tris-glycine SDS-PAGE gel. Proteins were transferred to 0.2-μm nitrocellulose membrane and the transferred membrane was blocked with 5% (*w/v*) nonfat dried milk in Tris-buffered saline with 0.1% Tween-20 (TBST; 10 mM Tris, 150 mM NaCl, pH 7.6) for 1 h at room temperature. After washing blocked membrane with PBS four times for 10 min, the membrane was incubated with the following primary antibodies at 4 °C for 16 h: APP (6E10; BioLegend, monoclonal, #803002), caveolin (cav-1; BD Transduction Laboratories, polyclonal, #610059), flotillin-1 (BD Transduction Laboratories, monoclonal, #610880), GAPDH (Cell Signaling Technology, Danvers, MA, USA, monoclonal, #14C10), β-actin (EnoGene, New York, NY, USA, monoclonal, #E12-041). Next, the membrane was washed with TBST four times and incubated with horseradish-peroxidase-conjugated goat antirabbit IgG (Invitrogen, polyclonal, #656120) or goat antimouse IgG (Invitrogen, polyclonal, #G21040) antibodies for 1 h at room temperature to detect each primary antibody. After incubation with the secondary antibody, the membrane was washed again with TBST four times. We used enhanced chemiluminescence reagent (Westsave, #LF-QC0101), and signals were captured with film (MTC Bio, Metuchen, NJ, USA, #A8815). The intensity of bands was captured by LAS-3000 system (Fuji Film) and analyzed by Multi Gauge V3.0.

### 4.9. The Localization of Surface APP in Lipid Raft Microdomains

To monitor the localization of cell surface APP between lipid raft and nonlipid raft microdomains, cells were incubated with PBS supplemented with 0.25 mg/mL sulfo-NHS-biotin for 10 min at 4 °C to label all cell surface proteins. Remaining biotins were washed away with 100 mM glycine in PBS three times. Then, cells were collected with 0.25% trypsin-EDTA and lysed in 4-morpholineethanesulfonic acid (MES)-buffered saline (MBS; 25 mM MES, 150 mM NaCl, pH 6.5) containing 500 mM sodium carbonate (Sigma, #S7795) and a protease inhibitor cocktail Set III (Calbiochem, San Diego, CA, USA, #535140). The lysates were homogenized 20 times with a 2 mL homogenizer and sonicated for 1 min (20 s sonication followed by 10 s interval). Cells were not homogenized with needle for this experiment. Equal amounts of protein were added to 0.8 mL of 80% (*w/v*) sucrose in MBS. Then, 1.6 mL of 35% (*w/v*) sucrose and 5% (*w/v*) sucrose in MBS were layered in a 5.1 mL ultracentrifuge tube (Beckman Coulter, Brea, CA, USA, #326819) to form a discontinuous sucrose gradient. The tubes were placed in a Beckman SW 55 Ti rotor (Beckman Coulter) and centrifuged at 50,000 rpm for 3 h at 4 °C. From top to bottom, 12 fractions (0.4 mL each) were collected. Fractions #4–6 were combined as lipid raft fractions and fractions #8–12 were combined as nonlipid raft fractions. Equal amounts of protein from lipid raft and nonlipid raft fractions were incubated with streptavidin-agarose slurry at 4 °C for 16 h to pull down biotin-labeled proteins. After washing, the biotin-labeled proteins were analyzed by Western blot to detect APP, β-actin, GAPDH, and the lipid raft markers caveolin and flotillin.

### 4.10. Rate of Endocytosis of Surface APP in Lipid Raft Microdomains

To monitor the contribution of lipid raft microdomains to APP endocytosis, cells were washed with ice-cold PBS to block protein trafficking at the surface level. All procedures were performed on ice. Cells were incubated with PBS supplemented with 0.25 mg/mL sulfo-NHS-SS-biotin for 10 min at 4 °C to label all surface proteins. Excess biotin was washed out, and cells were incubated with PBS containing 1% BSA for 15 min at 4 °C. After washing, cells were incubated at 37 °C for 10 min to allow internalization of biotin-labeled surface proteins. Then, cells were quickly placed on ice and washed with ice-cold PBS to stop internalization. Remaining cell surface biotin was removed by incubating cells twice with a reducing agent (50 mM sodium-2-mercapoethanesulfomate, 150 mM NaCl, 1 mM EDTA, 0.2% BSA, 20 mM Tris HCl, pH 8.6) for 25 min at 4 °C. Then, the reducing agent was quenched by ice-cold 5 mg/mL iodoacetamide in 1% BSA for 10 min. After washing, cells were harvested with 0.25% trypsin-EDTA. Cells were lysed with MBS containing 500 mM sodium carbonate with a protease inhibitor cocktail. The lysates were homogenized 20 times with 2 mL homogenizer followed by sonication for 1 min (20 s sonication followed by 10 s interval). Cells were not homogenized with needle for this experiment. After discontinuous sucrose gradient centrifugation, biotin-labeled internalized proteins were pulled down from fractions as described above. APP, β-actin, GAPDH, caveolin, and flotillin were monitored by Western blot.

### 4.11. Localization of APP in Early Endosomes

CHO PS1 WT and ΔE9 cells were grown on poly-D-lysine-coated glass cover slips. Surface APP was labeled with 6E10 antibody at 4 °C for 1 h, then, cells were transferred to 37 °C in order to allow internalization. Internalization was stopped with ice-cold PBS and cells fixed with 4% paraformaldehyde in 4% sucrose at room temperature for 15 min. After washing, cells were incubated with goat antimouse IgG secondary antibody to captured remaining surface APP, which eliminated the surface signal. Internalized APP was captured after permeabilization. Next, cells were permeabilized with 0.1% Triton X-100/2% BSA/PBS buffer for 5 min. Then, cells were blocked with 2% BSA in PBS for 1 h. Cells were incubated with an antibody to early endosome marker, EEA1 (Cell Signaling, monoclonal, #C45B10), in blocking buffer for 2 h at room temperature. Following PBS washes, cells were incubated with goat antimouse conjugated with Alexa Fluor 647 and goat antirabbit conjugated with Alexa Fluor 488 (Invitrogen, #A1S1034) secondary antibodies in blocking buffer overnight to detect internalized APP and early endosomes, respectively. The next day, cells were washed with PBS and mounted with mounting medium. Immunofluorescence staining was monitored on a confocal microscope (LSM710, Zeiss). The colocalization of APP and early endosomes were measured by Image J program.

### 4.12. Aβ42 Peptide ELISA Assay

CHO PS1 WT cells were pretreated with 0, 75, or 150 μM β-cholesterol for 1 h and CHO PS1 ΔE9 cells were pretreated with 0, 2, or 5 mM MβCD for 30 min. Then, cells were washed and replenished with fresh culture media for 2 h. Following incubation, 1 mL of culture media was collected and centrifuged at 12,000 rpm for 5 min to spin down cell debris. Aβ42 levels were measured using a High Sensitivity Human Amyloid β42 ELISA Kit (Millipore, #EZHS42).

### 4.13. Statistical Analysis

Data are expressed as mean ± SEM. We conducted statistical analysis using one-way ANOVA between the controls and the treated experimental groups; and considered *p* < 0.05 statistically significant.

## Supplementary Materials

The following are available online. Figure S1. Cellular cholesterol levels in CHO PS1 WT and PS1 ΔE9 cells; Figure S2. APP localization in cholera toxin B positive-raft microdomains was altered by cellular cholesterol in CHO PS1 WT and PS1 ΔE9 cells; Figure S3. The endocytosis rate of transferrin was not affected by elevated cellular cholesterol levels; Figure S4. APP endocytosis rate in PS1 WT cells was increased by increasing cellular cholesterol levels; Figure S5. Lipid raft fractionation; Figure S6. Surface APP localization in lipid raft microdomains was affected by the cholesterol levels in HeLa APPswe cells; Figure S7. Cellular cholesterol levels altered raft-dependent APP endocytosis in HeLa APPswe cells; Figure S8. Modulating cellular cholesterol levels regulated secreted Aβ42 levels in CHO PS1 WT and PS1 ΔE9 cells; Figure S9. Cellular cholesterol levels altered the accumulation of APP in early endosomes.
