# Supplementary material for: Preferred Endocytosis of Amyloid Precursor Protein from Cholesterol-Enriched Lipid Raft Microdomains
Source: Molecules. 2020 Nov 24;25(23):5490. doi: 10.3390/molecules25235490 (PMC7727664; doi:10.3390/molecules25235490)
Supplement: Supplementary file 1 [file molecules-25-05490-s001.pdf]

Preferred endocytosis of amyloid precursor protein from cholesterol-enriched lipid raft microdomains

Yoon Young Cho[#], Oh-Hoon Kwon[#], Sungkwon Chung*

Department of Physiology, Sungkyunkwan University School of Medicine,

Suwon 16419, South Korea

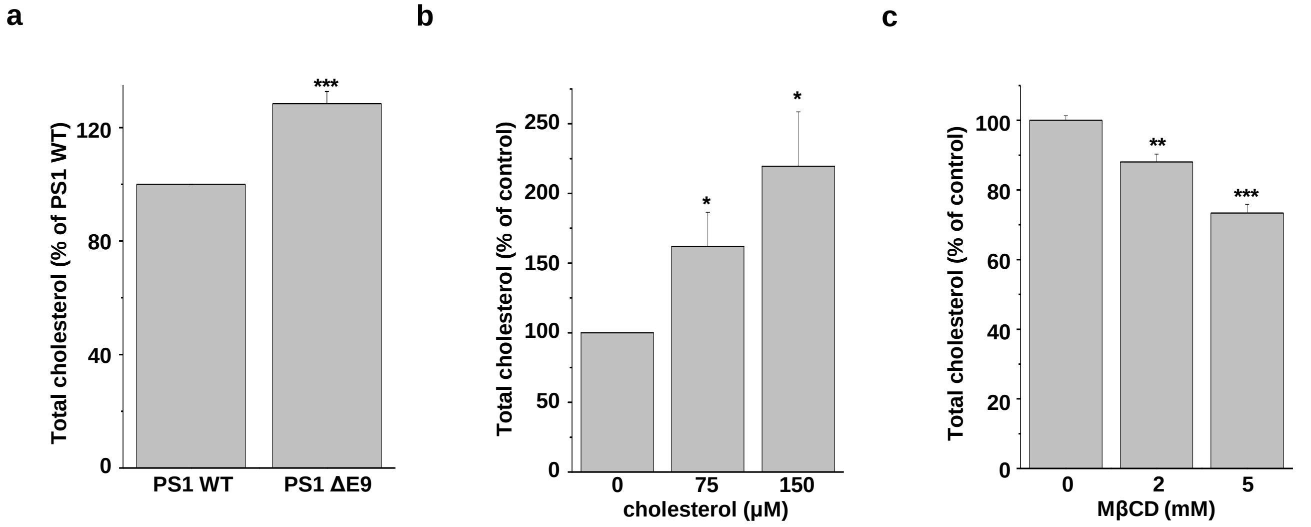

Supplemental figure S1. Cellular cholesterol levels in CHO PS1 WT and PS1 ΔE9 cells.
(a) Total membrane cholesterol was measured in CHO PS1 WT and PS1 ΔE9 cells (n=7) using Amplex Red Cholesterol Assay Kit (Invitrogen, #A12216). (b) CHO PS1 WT cells were pre-treated with 0, 75, or 150 μM β-cholesterol to increase cellular cholesterol levels (n=6), and (c) CHO PS1 ΔE9 cells were incubated with 0, 2, or 5 mM MβCD to decrease cellular cholesterol levels (n=6). Statistical analysis was carried out by one-way ANOVA: $^{*}p<0.05$, $^{**}p<0.01$, $^{***}p<0.001$.

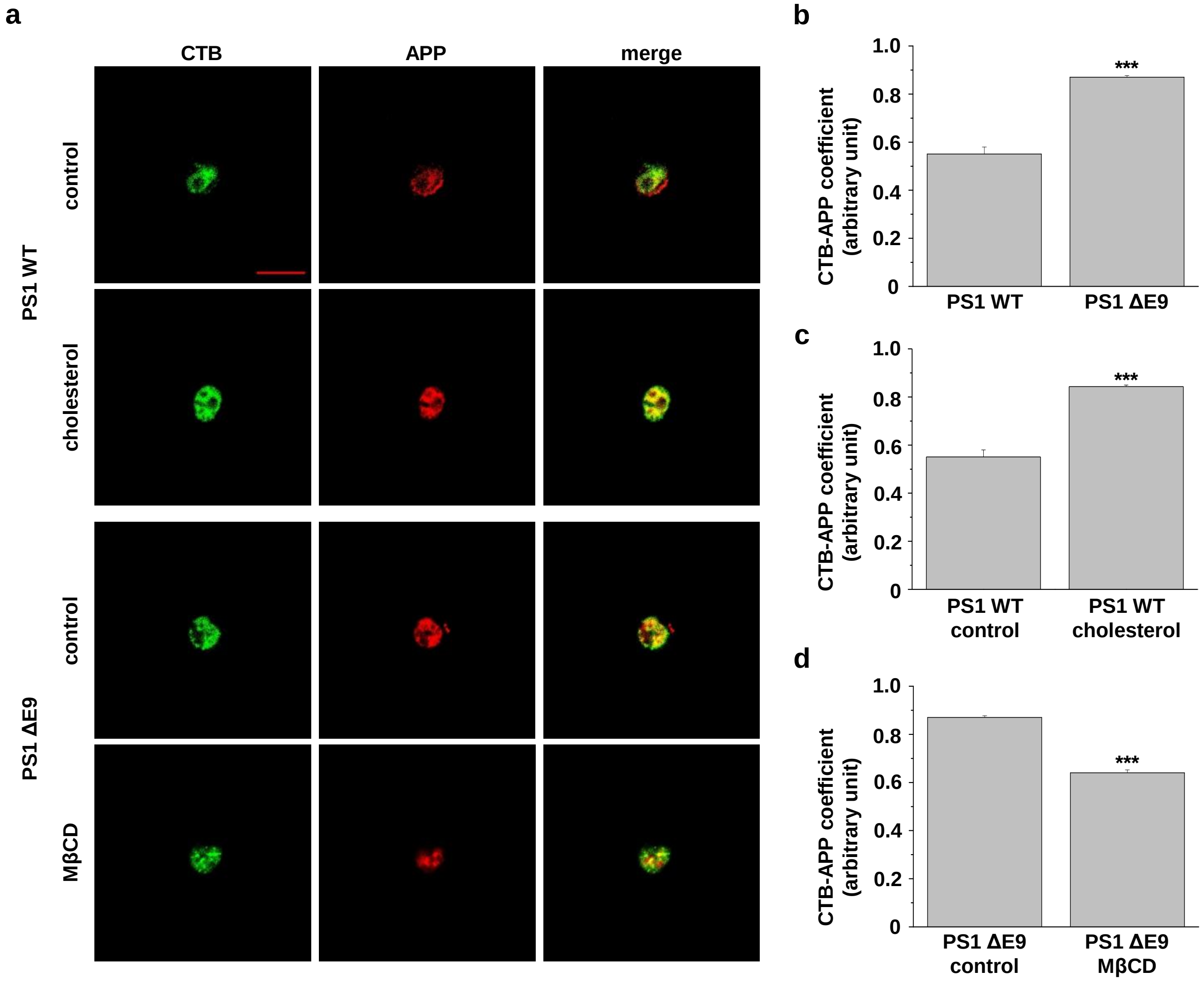

Supplemental figure S2. APP localization in cholera toxin B positive-raft microdomains was altered by cellular cholesterol in CHO PS1 WT and PS1 ΔE9 cells.

(a) CHO PS1 WT cells were incubated with 150 μM β-cholesterol and PS1 ΔE9 cells were treated with 5 mM MβCD. Then, cells were incubated with 6E10 antibody and 10 μg/ml of cholera toxin B (CTB) at 4°C to label surface APP and lipid raft microdomains, respectively. Representative confocal image demonstrated the co-localization of APP and CTB. Data are analyzed from five independent experiments. Scare bars correspond to 10 μm. The ratio of overlap APP over CTB are indicated for (b) CHO PS1 WT cells and PS1 ΔE9 (n=5), (c) PS1 WT control and cholesterol-treated PS1 WT cells (n=5), and (d) PS1 ΔE9 and MβCD-treated PS1 ΔE9 cells (n=5). The co-localization of APP and CTB was determined with Image J. Statistical analysis was carried out by one-way ANOVA: ***$p<0.001$.

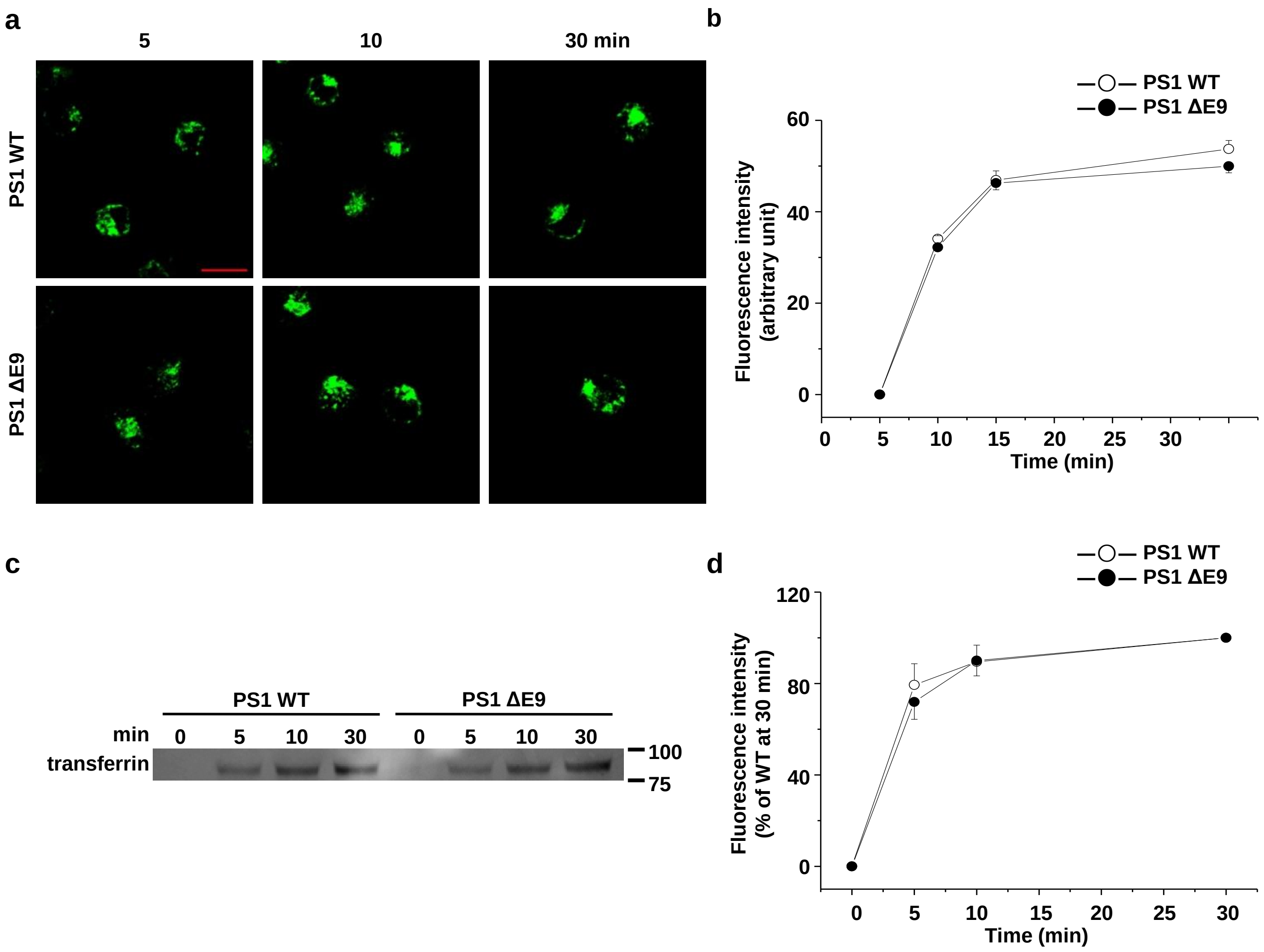

Supplemental figure S3. The endocytosis rate of transferrin was not affected by elevated cellular cholesterol levels.

(a) Cells were treated with Alexa488-conjugated transferrin in PBS at 37°C for indicated time periods to permit endocytosis. After removing remaining transferrin with acidic buffer, cells were fixed and visualized under a fluorescence microscope. Representative confocal image was from three independent experiments. Scare bars correspond to 10 μm. (b) Fluorescence intensities of internalized transferrin were analyzed using Image J software (n=3). (c) Cells were incubated with Alexa488-conjugated transferrin at 37°C for varying time periods to allow internalization. After washing, cells were harvested, and the same amount of protein was run on western blots (n=4). (d) Fluorescent bands were detected by a LAS-3000 system (Fuji Film, Japan) and were analyzed with the Multi Gauge software (n=4).

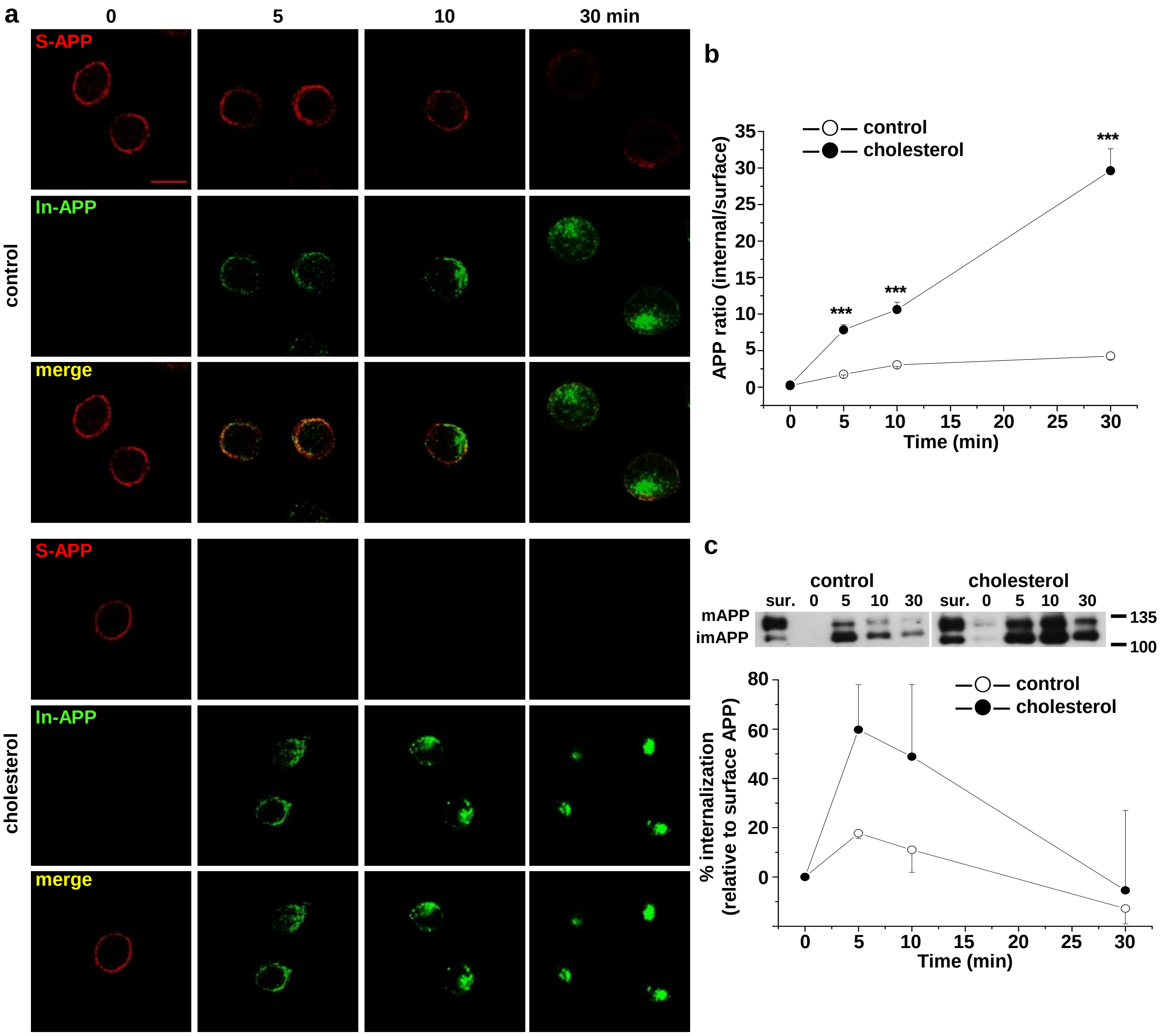

Supplemental figure S4. APP endocytosis rate in PS1 WT cells was increased by increasing cellular cholesterol levels.

CHO PS1 WT cells were pre-treated with 150 μM β-cholesterol to increase cellular cholesterol level. Cells were then labeled with APP antibody at 4°C to visualize APP endocytosis as described in Fig. 2. (a) Representative confocal images show the localization of APP at indicated time points from four independent experiments. Scare bars correspond to 10 μm. (b) APP endocytosis was measured as the ratio of internalized APP over surface APP (n=4). (c) APP endocytosis was quantified using EZ-Link sulfo-NHS-SS-biotin as described in Fig. 2. Total biotin-labeled APP (surface APP; sur.) was also detected. The upper panel shows a typical western blot result. The lower panel shows the rate of APP endocytosis by comparing internalized APP to surface APP (n=4). The band density was detected by a LAS-3000 system (Fuji Film, Japan) and analyzed with Multi Gauge software. Statistical analysis was carried out by one-way ANOVA: ***p<0.001.

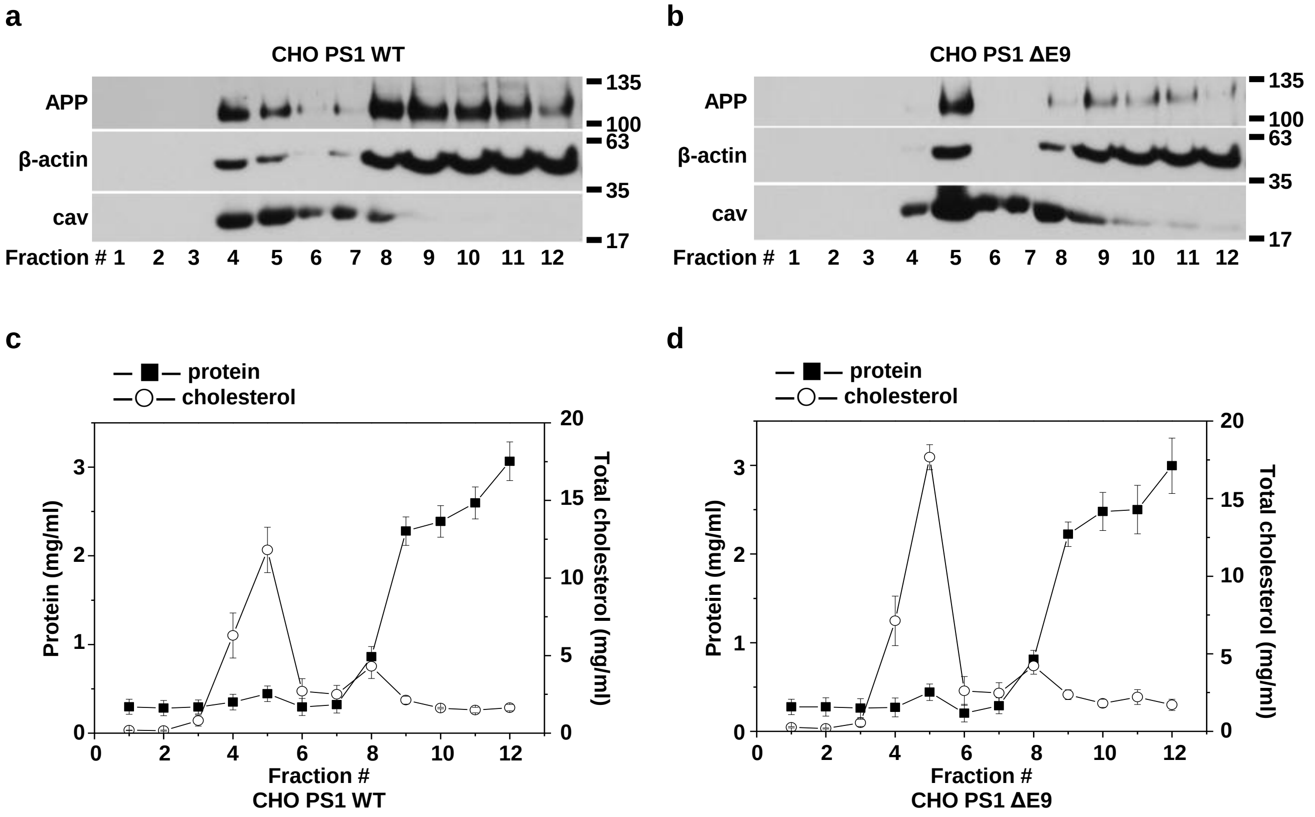

Supplemental figure S5. Lipid raft fractionation.
CHO PS1 WT and PS1 ΔE9 cells were collected with sodium carbonate buffer and fractionated with discontinuous sucrose density gradients as described in Methods. A total of 12 fractions were obtained from the top to the bottom, and equal volumes of each fraction were run on western blot to monitor the localization of total APP. A typical western blot image indicated localization of APP, β-actin, and caveolin (lipid raft marker) (n=5) within 12 fractions (a) from PS1 WT and (b) PS1 ΔE9 cells, respectively. (c) From PS1 WT and (d) PS1 ΔE9 cells, protein and cholesterol levels in each fraction were measured (n=5).

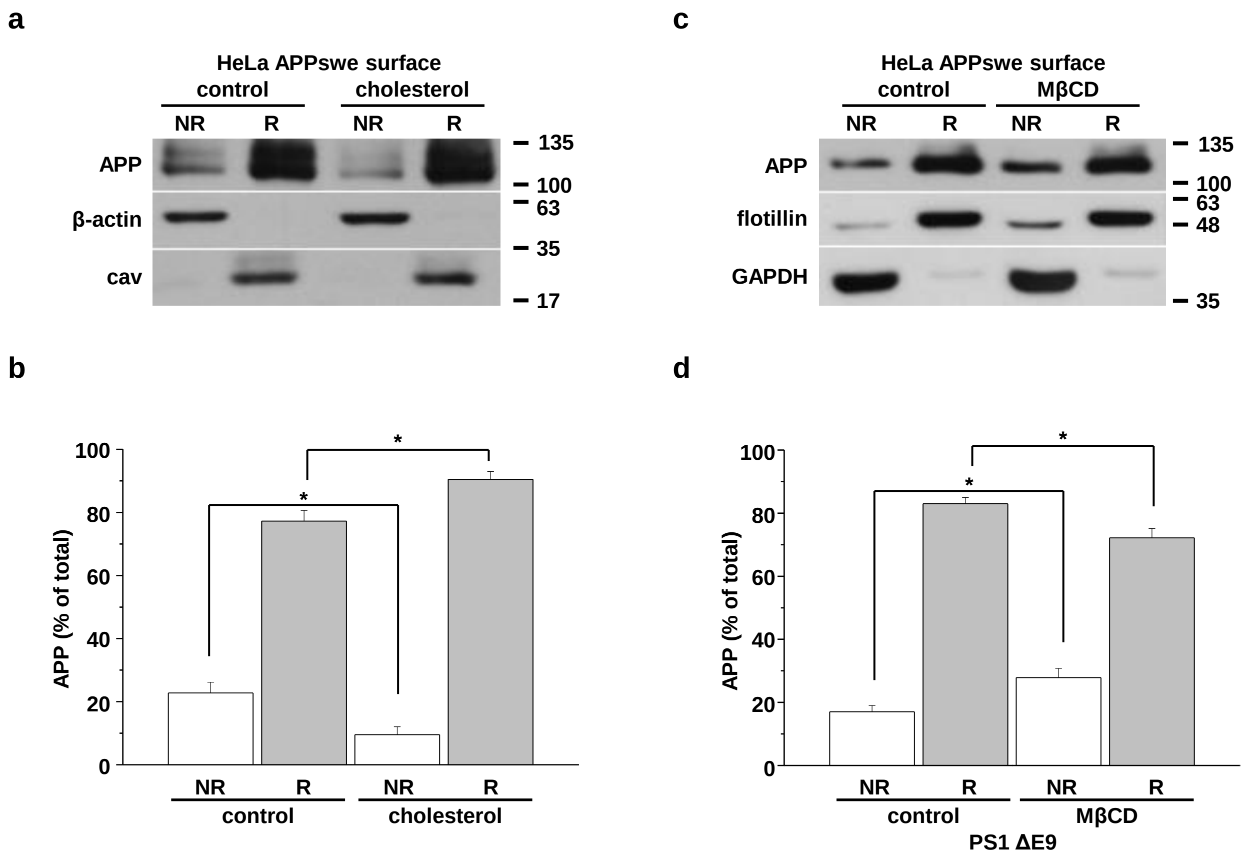

Supplemental figure S6. Surface APP localization in lipid raft microdomains was affected by the cholesterol levels in HeLa APPswe cells.

Surface proteins were labeled with biotin at 4°C before fractionation. Lipid rafts (4 to 6, R) or non-lipid rafts (8 to 12, NR) were obtained as described in Fig. 4. Then, the same amount of biotin-labeled proteins was captured with streptavidin beads, and captured biotin-labeled proteins were run on western blots to detect APP, β-actin, GAPDH, flotillin, and caveolin. (a) HeLa APPswe cells were pre-treated with 150 μM β-cholesterol before labeling all surface proteins with EZ-Link NHS-biotin. The representative western blot image indicates the localization of surface APP in lipid raft and non-lipid raft fractions (n=5). (b) Relative band densities show the ratio of surface APP from raft and non-raft fractions (n=5). (c) HeLa APPswe cells were pre-incubated with 1 mM MβCD before labeling all surface proteins and fractionation as described in Fig. 4. Representative western blot result demonstrated biotin-labeled surface APP localization from lipid raft and non-lipid raft fractions (n=4). (d) The ratio of surface APP from each fractions was analyzed (n=4). Statistical analysis was carried out by one-way ANOVA: *$p<0.05$.

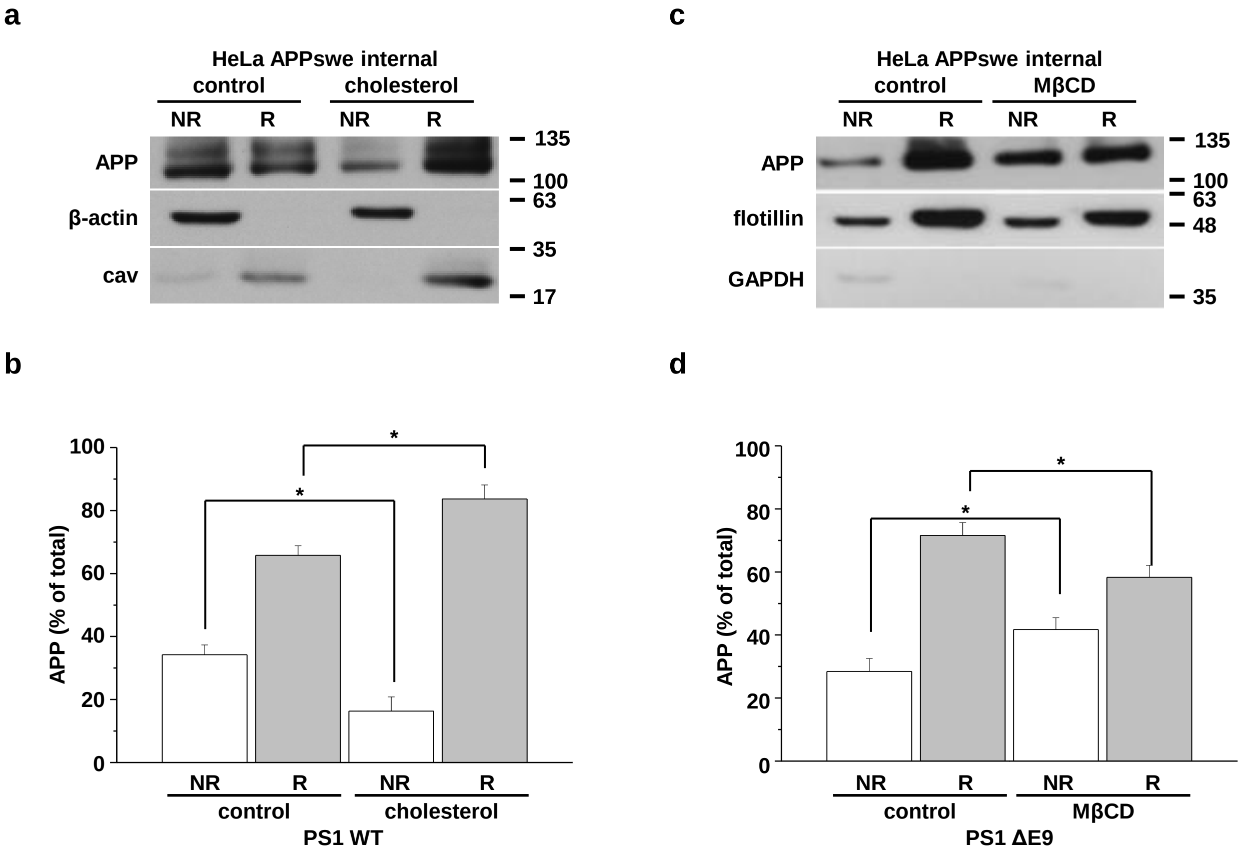

Supplemental figure S7. Cellular cholesterol levels altered raft-dependent APP endocytosis in HeLa APPswe cells.

To verify raft-induced APP endocytosis, cells were biotinylated at 4°C to label surface proteins. Then, cells were transferred to 37°C for 10 min to allow internalization of biotin-labeled surface proteins, followed by fractionation as described in Fig. 5. After fractionation, the equal amount of biotin-labeled protein from fractions 4-6 (lipid rafts, R) or 8-12 (non-lipid rafts, NR) was captured with streptavidin beads. Internalized biotin-labeled proteins were run on western blotting. (a) HeLa APPswe cells were pre-treated with 150 μM β-cholesterol before fractionation. The corresponding western blot result shows the localization of biotin-labeled internalized APP (n=5). (b) The relative band density indicates the ratio of internalized APP from lipid raft and non-lipid raft fractions (n=5). (c) HeLa APPswe cells were incubated with 1 mM MβCD. Before fractionation, all surface proteins were labeled and internalized for 10 min as described in Fig. 5. Representative western blot results demonstrate internalized APP levels from raft and non-raft fractions (n=5). (d) The band densitometry of internalized APP from lipid raft and non-lipid raft fractions was analyzed (n=5). Statistical analysis was performed by one-way ANOVA: *p<0.05.

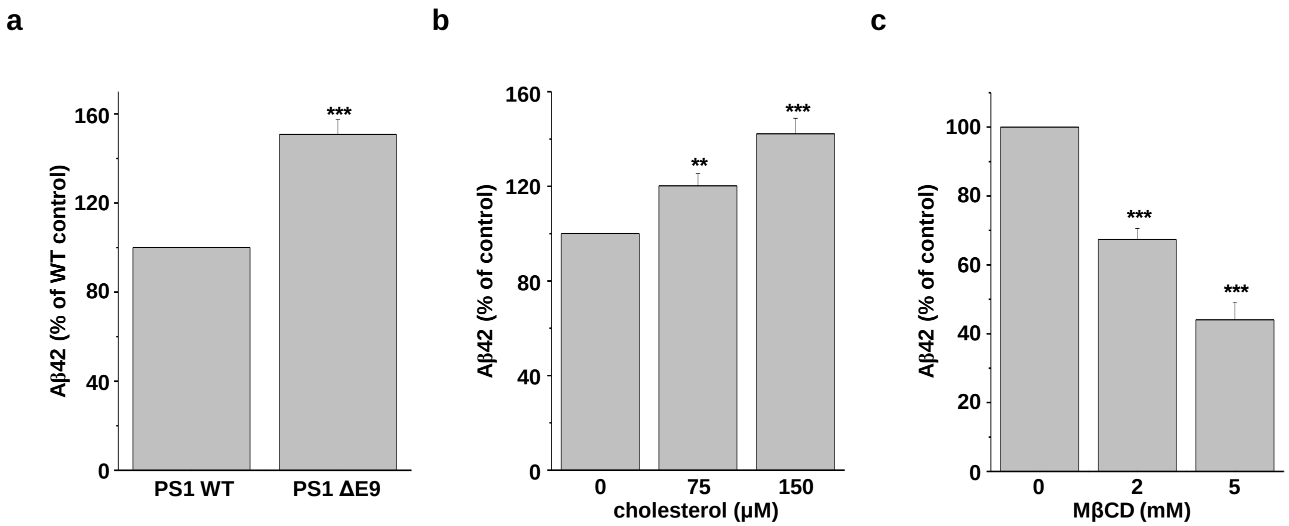

Supplemental figure S8. Modulating cellular cholesterol levels regulated secreted Aβ42 levels in CHO PS1 WT and PS1 ΔE9 cells.
(a) Aβ42 levels were measured from the conditioned media in CHO PS1 WT and PS1 ΔE9 cells using Aβ42 specific ELISA kit (n=5). (b) CHO PS1 WT cells were incubated with 0, 75, or 150 μM β-cholesterol. Then, cells were washed and replenished with conditioned media for 2 h. Aβ42 levels were analyzed from the conditioned media (n=4). (c) CHO PS1 ΔE9 cells were pre-treated with 0, 2, or 5 mM MβCD, and then cells were refreshed with new conditioned media for 2 h. Levels of Aβ42 were measured from conditioned media (n=4). Statistical analysis was performed by one-way ANOVA: $^{**}p<0.01$, $^{***}p<0.001$.

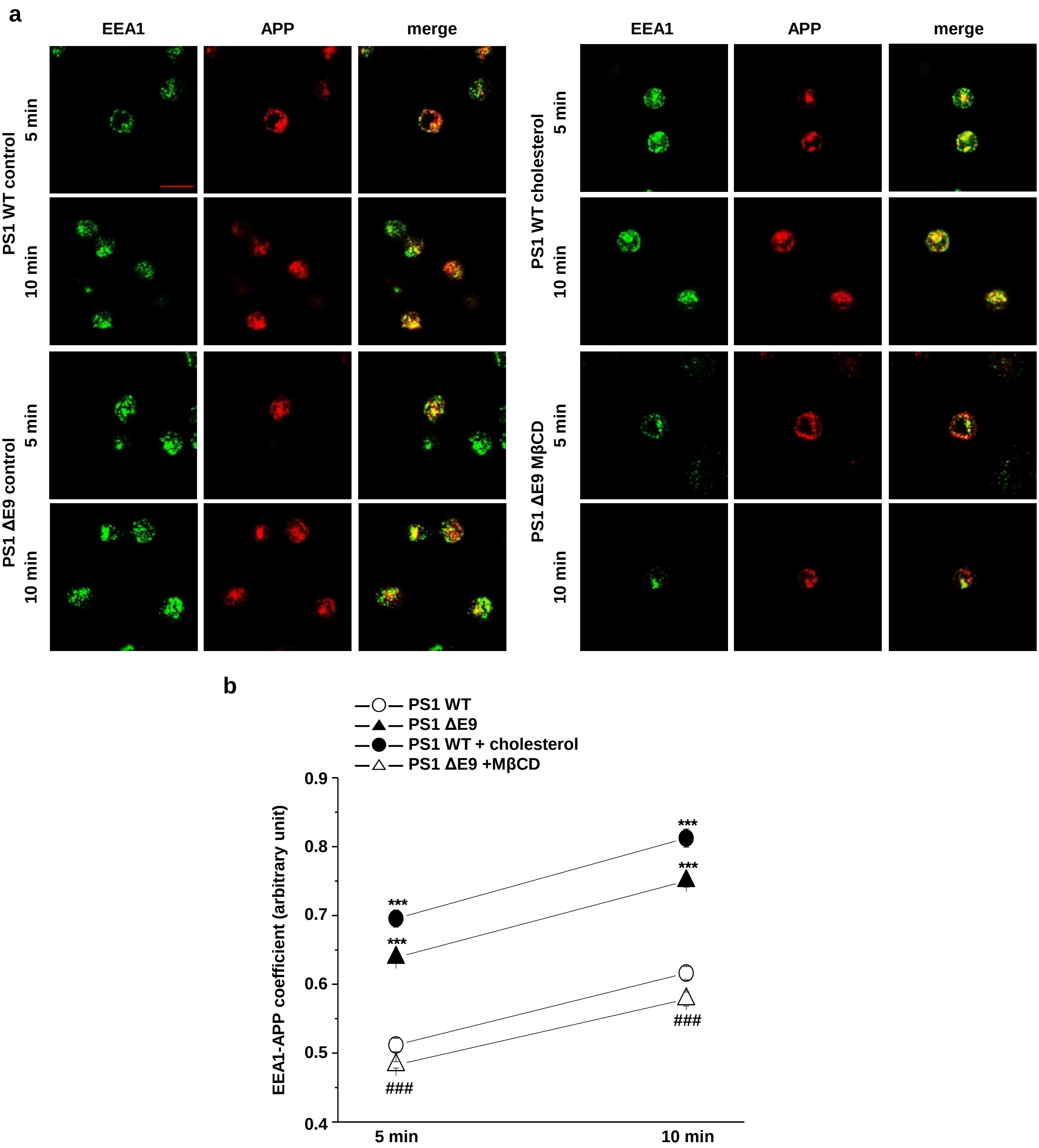
a
EEA1
APP
merge
PS1 WT control
5 min
10 min
PS1 ΔE9 control
PS1 WT cholesterol
PS1 ΔE9 MβCD
b
PS1 WT
PS1 ΔE9
PS1 WT + cholesterol
PS1 ΔE9 +MβCD
EEA1-APP coefficient (arbitrary unit)
0.9
0.8
0.7
0.6
0.5
0.4
***
###

Supplemental figure S9. Cellular cholesterol levels altered the accumulation of APP in early endosomes. CHO PS1WT cells were pre-treated with 150 μM β-cholesterol and PS1 ΔE9 cells were pre-treated with 5 mM MβCD. Cells were incubated with APP antibody at 4°C and transferred to 37°C for indicated times to allow internalization of the labeled surface APP. Then, cells were fixed and surface APP was stained with anti-mouse IgG secondary antibody to eliminate remaining surface APP signal. Following permeabilization, cells were labeled with EEA1 antibody to label early endosomes. Subsequently, anti-mouse Alexa647 (red)- or anti-rabbit Aexa488 (green)-conjugated secondary antibodies were used to label internalized APP and early endosomes, respectively. (a) Representative confocal image showing APP localization at each time point. Data are representative of four independent experiments. Scare bars correspond to 10 μm. (b) Fluorescence intensities of APP and EEA1 were measured using Image J. The co-efficiency of APP and early endosomes was determined with Image J (n=4). Statistical analysis was analyzed by one-way ANOVA: *** indicates $P<0.001$ significant difference compared to PS1 WT cells. ### represents $P<0.001$ significant difference compared to PS1 ΔE9 cell.
